# Antiarrhythmic Sotalol, Occlusion/Occlusion-like Syndrome in Rats, and Stable Gastric Pentadecapeptide BPC 157 Therapy

**DOI:** 10.3390/ph16070977

**Published:** 2023-07-07

**Authors:** Ivica Premuzic Mestrovic, Ivan Maria Smoday, Luka Kalogjera, Ivan Krezic, Helena Zizek, Hrvoje Vranes, Vlasta Vukovic, Katarina Oroz, Ivan Skorak, Ivan Brizic, Klaudija Hriberski, Luka Novosel, Ivana Kavelj, Ivan Barisic, Lidija Beketic Oreskovic, Slavica Zubcic, Sanja Strbe, Tomislav Mestrovic, Predrag Pavic, Mario Staresinic, Anita Skrtic, Alenka Boban Blagaic, Sven Seiwerth, Predrag Sikiric

**Affiliations:** 1 Department of Pharmacology, School of Medicine University of Zagreb, 10000 Zagreb, Croatia; premuzici@yahoo.com (I.P.M.); ivansmoday1@gmail.com (I.M.S.); lkalogjera9@gmail.com (L.K.); ivankrezic94@gmail.com (I.K.); zizekhelena@gmail.com (H.Z.); hrvoje.vranes@gmail.com (H.V.); vukovic.vlasta1@gmail.com (V.V.); oroz.kat@hotmail.com (K.O.); ivan.skorak@gmail.com (I.S.); 2360999@gmail.com (I.B.); klaudija.hriberski@gmail.com (K.H.); novosel0701@gmail.com (L.N.); ivana.kavelj@gmail.com (I.K.); inbarisic@gmail.com (I.B.); lidijabeketicoreskovic@gmail.com (L.B.O.); slavica.zubcic.kristo@bolnica-zadar.hr (S.Z.); strbes@gmail.com (S.S.); abblagaic@mef.hr (A.B.B.); 2 Department of Surgery, School of Medicine University of Zagreb, 10000 Zagreb, Croatia; mestrovic.tomislav@gmail.com (T.M.); p.d.pavic@gmail.com (P.P.); 3 Department of Pathology, School of Medicine University of Zagreb, 10000 Zagreb, Croatia; sven.seiwerth@mef.hr

**Keywords:** antiarrhythmic sotalol, occlusion/occlusion-like syndrome, stable gastric pentadecapeptide BPC 157, therapy, rats

## Abstract

We focused on the first demonstration that antiarrhythmics, particularly class II and class III antiarrhythmic and beta-blocker sotalol can induce severe occlusion/occlusion-like syndrome in rats. In this syndrome, as in similar syndromes with permanent occlusion of major vessels, peripheral and central, and other similar noxious procedures that severely disable endothelium function, the stable gastric pentadecapeptide BPC 157-collateral pathways activation, was a resolving therapy. After a high dose of sotalol (80 mg/kg intragastrically) in 180 min study, there were cause-consequence lesions in the brain (swelling, intracerebral hemorrhage), congestion in the heart, lung, liver, kidney, and gastrointestinal tract, severe bradycardia, and intracranial (superior sagittal sinus), portal and caval hypertension, and aortal hypotension, and widespread thrombosis, peripherally and centrally. Major vessels failed (congested inferior caval and superior mesenteric vein, collapsed azygos vein). BPC 157 therapy (10 µg, 10 ng/kg given intragastrically at 5 min or 90 min sotalol-time) effectively counteracted sotalol-occlusion/occlusion-like syndrome. In particular, eliminated were heart dilatation, and myocardial congestion affecting coronary veins and arteries, as well as myocardial vessels; eliminated were portal and caval hypertension, lung parenchyma congestion, venous and arterial thrombosis, attenuated aortal hypotension, and centrally, attenuated intracranial (superior sagittal sinus) hypertension, brain lesions and pronounced intracerebral hemorrhage. Further, BPC 157 eliminated and/or markedly attenuated liver, kidney, and gastrointestinal tract congestion and major veins congestion. Therefore, azygos vein activation and direct blood delivery were essential for particular BPC 157 effects. Thus, preventing such and similar events, and responding adequately when that event is at risk, strongly advocates for further BPC 157 therapy.

## 1. Introduction

We focused on the first demonstration of the severe occlusion/occlusion-like syndrome induced by antiarrhythmics, particularly class II and class III antiarrhythmic and beta-blocker sotalol, which is widely used [1,2,3]. On the other hand, we recently reviewed the cytoprotective stable gastric pentadecapeptide BPC 157 in resolving arrhythmias issues [4,5,6,7]. Beneficial action on striated, smooth, and heart muscle results in a therapeutic effect on heart disturbances, myocardial infarction, heart failure, pulmonary hypertension, arrhythmias, and thrombosis presentation [4,5,6,7].

Thereby, as before in the counteraction of the other occlusion/occlusion-like syndromes [8,9,10,11,12,13,14,15,16,17,18,19,20,21,22], we suggested the pentadecapeptide BPC 157 as the resolving therapy. While sotalol may appear with the additional injurious course (i.e., disabled beta-adrenergic system), the BPC 157 application should maintain a special therapy effect (i.e., activation of collateral pathways, such as azygos vein) recently especially reviewed [4,5,6,7] in the rescuing of all of the described occlusion/occlusion-like syndromes. In support, this beneficial effect (“bypassing key”) resolved a wide range of occlusion/occlusion-like syndromes vessels and multiple organ failures [8,9,10,11,12,13,14,15,16,17,18,19,20,21,22]. The counteracting effect on occlusion/occlusion-like syndrome occurred whatever the noxious cause that severely impairs endothelium function [11,12,13,16,17,18,19,20,21,22]. As therapy, it counteracted occlusion/occlusion-like syndromes provoked by a more non-specific challenge, i.e., absolute alcohol intragastric application [17], bile duct ligation acute pancreatitis [20], and intra-abdominal hypertension, grade III and grade IV [21]. Likewise, it rapidly acts against either of specific injuries (i.e., particular major vessel occlusion, peripheral or central [11,12,13,16], application of agents acting as adrenergic system (over)-stimulants, or particular receptors blockers, isoprenaline [22], neuroleptics, domperidone, and amphetamine application [19]). These were thought a crucial chain of events, essential vascular cytoprotective response rapidly upgrading the endothelium and minor vessels function as collateral pathways were activated depending on the injury [4,5,6,7] in particular, combined with attenuated/eliminated arrhythmias. Whatever the arrhythmia type [11,12,13,16,17,18,19,20,21,22], there was counteraction along with BPC 157 therapy azygos vein direct blood flow delivery and subsequent counteraction of occlusion/occlusion-like syndrome in rats [4,5,6,7].

Accordingly, given sotalol in a high dosage, one may perceive the full extent of the occlusion/occlusion-like syndrome due to the particular class II and class III antiarrhythmic and beta-receptor blockade extreme effects already in quite immediate post-application time. Tightly interconnected, rapidly occurring various arrhythmias courses may be fully correlated with the presentation of the closely interconnected multiple organ lesions [8,9,10,11,12,13,14,15,16,17,18,19,20,21,22]. Vice versa, the effect of therapy may be also correlated with the counteraction of particular lesions. Without BPC 157 therapy, there was an undisturbed cause-consequence lesions course. Brain presented with intracerebral and intraventricular hemorrhage, congestion and infarctions in the heart, hemorrhage in the lung, congestion in the liver, kidney, and gastrointestinal tract, arrhythmias of various types, and blood pressure disturbances (intracranial (superior sagittal sinus), portal and caval hypertension, and aortal hypotension), and widespread thrombosis, peripherally and centrally [8,9,10,11,12,13,14,15,16,17,18,19,20,21,22]. Major vessels failed (congested inferior caval and superior mesenteric vein, collapsed azygos vein) [8,9,10,11,12,13,14,15,16,17,18,19,20,21,22]. Therefore, occlusion/occlusion-like syndrome rapidly progresses pleiotropically cause-consequence course [8,9,10,11,12,13,14,15,16,17,18,19,20,21,22] all together unable to re-establish blood flow. Progressing endothelium dysfunction, progressing inability of minor vessels to adapt to taking over the function of disabled major vessels, and progressing arrhythmias concurrently occurred [8,9,10,11,12,13,14,15,16,17,18,19,20,21,22]. Therefore, these would appear as the particular disabilities of class II and class III antiarrhythmic effects and beta-receptor blockade in the sotalol-occlusion/occlusion-like syndrome.

These class II and class III antiarrhythmic effects and beta-receptor blockade in the sotalol-occlusion/occlusion-like syndrome specific points should be combined with isoprenaline, neuroleptics, domperidone, and amphetamine application, myocardial congestion, infarction, and re-infarction, and arrhythmias in occlusion/occlusion-like syndrome more specific injuries [11,12,13,16,19,22]. Likely, they may together show the disturbed beta-adrenergic system in a similar shared common occlusion/occlusion-like syndrome [22]. We recently evidenced in potential of the BPC 157 therapy the counteraction of a similar shared common occlusion/occlusion-like syndrome induced and shared by a disturbed dopamine system, by either dopamine antagonist (neuroleptics or domperidone) or dopamine agonist (amphetamine) [19]. In analogy, along with isoprenaline beta-agonist-induced myocardial infarction and reinfarction [22], there may be a similar complex noxious effect to the comparable occlusion/occlusion-like syndrome in the sotalol-challenged rats. Noteworthy, it may be also suggested that such sotalol-occlusion/occlusion-like syndrome would be properly antagonized by BPC 157 therapy. Illustrating the effect on bradyarrhythmias may be that the BPC 157 antidysrhythmic effect (along with rapid activation of the collateral pathway, i.e., azygos vein direct blood delivery) might resolve the otherwise deadly course of the occlusion/occlusion-like syndrome as a whole in the rats with the maintained intra-abdominal hypertension, grade III and grade IV [21]. The nodal rhythm, with dominant ST-elevation and bradycardia [21] otherwise resulting in extreme bradycardia and asystole as the ultimate outcome was resolved [21].

Likewise, given the innate pleiotropic effect of cytoprotection agents [4,5,6,7] that, as the concept holds [23,24], may protect endothelium, therapy by BPC 157 application (activation of collateral pathways depending on the injury, i.e., azygos vein direct, blood flow delivery), peripheral-central and central-peripheral link, may have an even more prominent cytoprotection background [4,5,6,7]. As a practical point (permitting also application via per-oral route), BPC 157 as a peptide native and stable in human gastric juice may continuously act as a cytoprotection mediator [4,5,6,7]. Note, used in ulcerative colitis trials, lethal dose (LD1) not achieved in toxicology studies [4,5,6,7]; BPC 157 therapy counteracts leaky gut as a stabilizer of cellular junctions [25] and acts as a free radical scavenger [25,26,27,28,29,30], in particular in vascular occlusion/occlusion-like studies [8,9,10,11,12,13,14,15,16,17,18,19,20,21,22]. As such, it may rapidly rescue endothelial function, and rapidly adapt minor vessels for taking over the function of disabled major vessels, and recruit collateral pathways, so that the arrhythmias cause-consequences and occlusion/occlusion-like syndrome would be rapidly reversed [8,9,10,11,12,13,14,15,16,17,18,19,20,21,22]. Besides, there was a particular interaction with the NO-system as a whole (NO-release of its own, modulation (counteraction) of the effects of both NOS-blockade (L-NAME hypertension, pro-thrombotic effect) and NOS-over-activity (L-arginine hypotension, anti-thrombotic effect) [31,32,33,34,35]. Particular controlling of vasomotor tone was evidenced through the activation of the Src-Caveolin-1-eNOS pathway [36,37]. There was the innate maintenance of the function of thrombocytes (without interference with coagulation) [35,38,39]. These were along with the interaction with other molecular pathways [25,30,36,37,40,41,42,43,44,45,46,47].

Therefore, we considered disputed sotalol [1,2,3] (increased mortality in the SWORD trial [48]), depressed myocardial contractility, and cardiac output, and negatively affected myocardial performance [49,50] in relation to shared findings on the occlusion/occlusion-like syndrome [8,9,10,11,12,13,14,15,16,17,18,19,20,21,22] (especially isoprenaline-induced myocardial infarction and reinfarction) [22], and we challenged these arrhythmias pathways with the sotalol high dose [50] application. Such a complex effect may reconcile several seemingly opposite points in the sotalol issue (i.e., prolongation of the plateau duration increases the effective refractory period (decreased incidence of re-entry) vs. prolongation of the plateau duration increases the likelihood of developing early afterdepolarizations and torsades de pointes) toward the comparable occlusion/occlusion-like syndrome. First, since long ago, as pointed out by [51], sotalol is an effective antiarrhythmic in various animal models of arrhythmia (e.g., chloroform, hydrocarbon-catecholamine, ouabain, and coronary ligation initially in canine models) [51,52,53,54,55,56]. However, in general, there may be a significant reduction in the sympathoadrenal basal activity [57] (i.e., binding to the peripheral β-adrenoceptors may prevent catecholamines to exert their action), but a slow-down in heart pumping and heartbeats. There is a strong anti-arrhythmic effect (i.e., supraventricular arrhythmias, recurrent atrial fibrillation) long ago demonstrated [58], but also severe prolonged QTc interval prolongation, life-threatening ventricular arrhythmias, including polymorphous ventricular tachycardia or torsade de pointes [58] (while blood not properly pumped out of the heart, and thereby, pooling and forming a clot [8]). Long ago, there was a thrombolytic effect of beta blockers and sotalol, in particular [59], beta-blockers improve endothelial function [60], but sotalol-induced vasculitis [61] and diffuse intra-alveolar hemorrhage [62]. 

Note, with respect to the occlusion/occlusion-like syndrome course, these all should converge to the arterial and venous thrombosis occurring peripherally and centrally [8,9,10,11,12,13,14,15,16,17,18,19,20,21,22], and harmfully implicating the failed major vessels [8,9,10,11,12,13,14,15,16,17,18,19,20,21,22]. Therefore, congested inferior caval vein, superior mesenteric vein, and collapsed azygos vein (no direct blood flow, no rescuing pathway) indicated toward rapidly acting Virchow triad circumstances [8,9,10,11,12,13,14,15,16,17,18,19,20,21,22] in occlusion/occlusion-like syndromes whatever the cause [4,5,6,7]. Finally, in counteraction of the occlusion/occlusion-like syndrome [8,9,10,11,12,13,14,15,16,17,18,19,20,21,22], the resolution by BPC 157 therapy may provide a contention that might bring into the cytoprotection issue and novel common therapy solution also the other arrhythmias and disturbances [4,5,6,7]. These were those induced by digitalis [63], hyperkalemia [64], hypokalemia [65], succinylcholine [66], bupivacaine [67], lidocaine [68], and neuroleptics [69]) and congestive heart failure and increased big endothelin-1 plasma concentration [70] that BPC 157 might counteract. 

Concluding, intragastric application of the stable gastric pentadecapeptide BPC 157 (i.e., as a part of its stomach cytoprotection background [4,5,6,7]) was given shortly after intragastric application of the sotalol, or much later, in the advanced course of the sotalol bradycardias. Likely, this would show BPC 157 beneficial therapeutic effect in the counteraction of the full occlusion/ occlusion-like syndrome that may have a particular class III arrhythmic and beta-blockade background in rats. 

## 2. Results

We revealed that the application of sotalol also provoked vascular failure and a perilous syndrome occurring peripherally and centrally. It might be a highly noxious syndrome, similar to those occlusion/occlusion-like syndromes previously described after major vessel occlusion (occlusion syndromes) as well as other alike noxious procedures (occlusion-like syndromes) [8,9,10,11,12,13,14,15,16,17,18,19,20,21,22].

The BPC 157 therapy effect was comparable to the previous BPC 157 therapy efficacy noted in the mentioned occlusion/occlusion-like syndromes [8,9,10,11,12,13,14,15,16,17,18,19,20,21,22]. This might be the attenuated/counteracted intracranial (superior sagittal sinus) hypertension and aortal hypotension, major ECG disturbances, progressing arterial and vein thrombosis, lesions in the brain, heart, lungs, liver, kidneys, and gastrointestinal tract [8,9,10,11,12,13,14,15,16,17,18,19,20,21,22]. The key effect of BPC 157 treatment in sotalol rats might be as before in the recovery of the occlusion and occlusion-like syndromes. The rescuing activation of the azygos vein and the prompt and sustained direct blood delivery from the inferior caval vein to the superior caval vein might be responsible for the instant breaking of the vicious cycle [8,9,10,11,12,13,14,15,16,17,18,19,20,21,22].

### 2.1. A Perilous Syndrome Occurred Peripherally and Centrally

#### 2.1.1. Blood Pressure Disturbances

**Figure 1:**
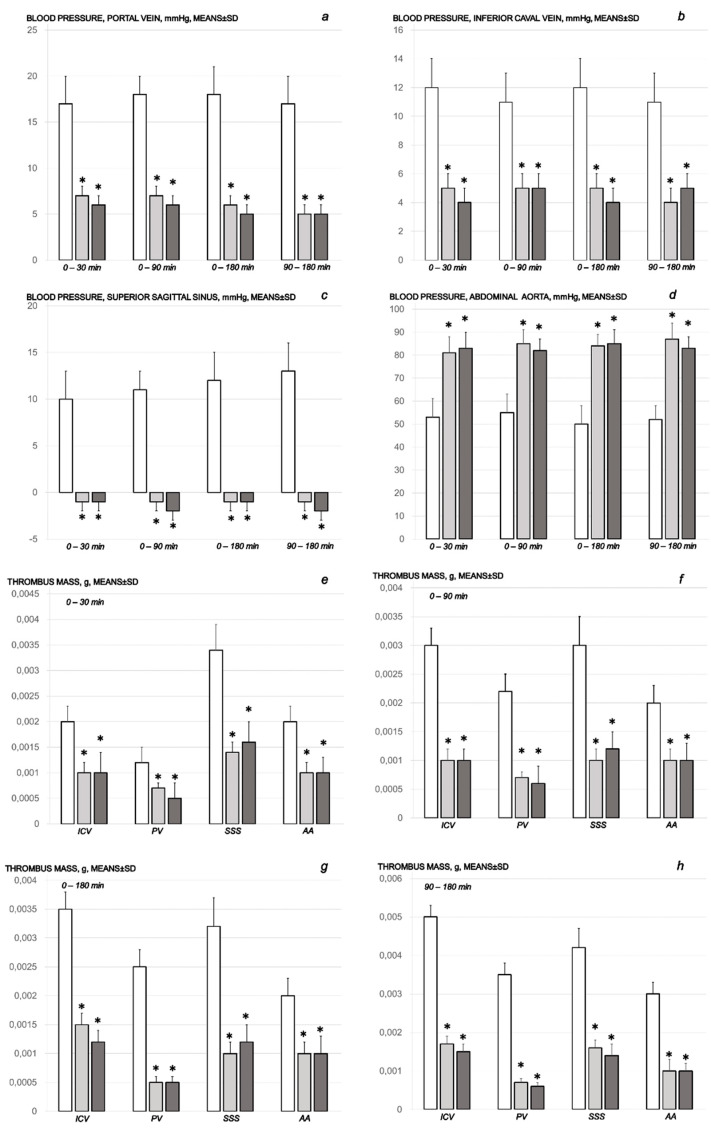
Blood pressure assessment (***a–d***), and thrombosis development (***e–h***). Portal (***a***), caval (***b***), and intracranial (superior sagittal sinus) (***c***) hypertension, and aortal hypotension (***d***) in mmHg; thrombosis development (***e****–**h***) in the inferior caval vein (***ICV***), portal vein (***PV***), superior sagittal sinus (***SSS***), and abdominal aorta (***AA***). Assessment at the end of the sotalol-time period of 15 min, 90 min, and 180 min. Therapy included BPC 157 (10 μg/kg (light gray bars) or 10 ng/kg (dark gray bars)) or saline (5 mL/kg) (white bars). They were given as an intragastric administration at 5 min following sotalol for assessment at 15 min (***e***), 90 min (***f***), and 180 min (***g***) sotalol-time. As a delayed regimen (***h***), the therapy was given as an intragastric administration at 90 min sotalol-time for assessment at 180 min sotalol-time. Means ± SD, * *p* ˂ 0.05, at least, vs. control.

As the lesion spread more and more, the severity might be illustrated both peripherally (portal and caval hypertension, aortal hypotension) as well as centrally (superior sagittal sinus hypertension) (Figure 1). Perceived as a cause-effect relation that should be essentially affected by the therapy application, BPC 157 rapidly reduced blood pressure disturbances that were induced by sotalol application, both those short-lasting (given BPC 157 application at 5 min sotalol-time) and those long-lasting (given BPC 157 application at 90 min sotalol-time). Consistently with a prominent therapy effect, both peripherally and centrally, BPC 157 application eliminated, or at least markedly attenuated, the portal and caval hypertension and intracranial (superior sagittal sinus) hypertension as well as the aortal hypotension.

#### 2.1.2. Thrombosis

Likewise, from the very beginning, as noted already at 15 min, the cause-effect course of the therapy might be the fact that BPC 157 reduced thrombosis, both peripherally (i.e., portal vein, inferior caval vein, and abdominal aorta) and centrally (i.e., superior sagittal sinus) (Figure 1). An alike effect occurred with BPC 157 application given at 90 min sotalol-time. Without therapy, the progressed thrombosis was sustainably present in arteries and veins.

#### 2.1.3. Collateral Pathways, Blood Vessels, and Brain Gross Presentation

**Figure 2:**
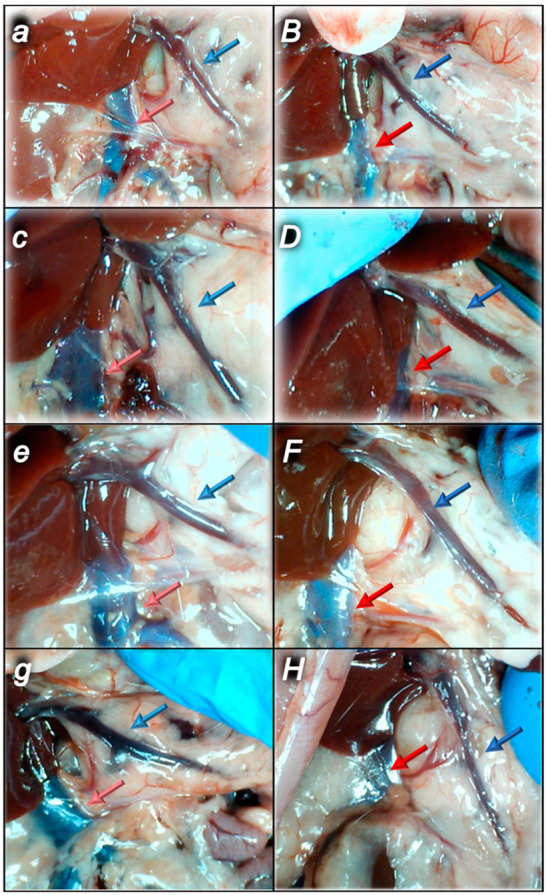
Illustrative presentation of the superior mesenteric vein (blue arrows) and inferior caval vein (red arrows) in the sotalol control rats (small letters, light arrows) and in BPC 157-treated rats (capitals, dark arrows) assessed at 15 min (***a***,***B***), 90 min (***c***,***D***) and 180 min (***e***,***F***,***g***,***H***) following application of sotalol. Medication was given as an early application at 5 min after sotalol (***a***,***B***,***c***,***D***,***e***,***F***), or as delayed post-treatment (***g***,***H***) (at 90 min and assessment at 180 min sotalol-time), saline or BPC 157 therapy given intragastrically.

**Figure 3:**
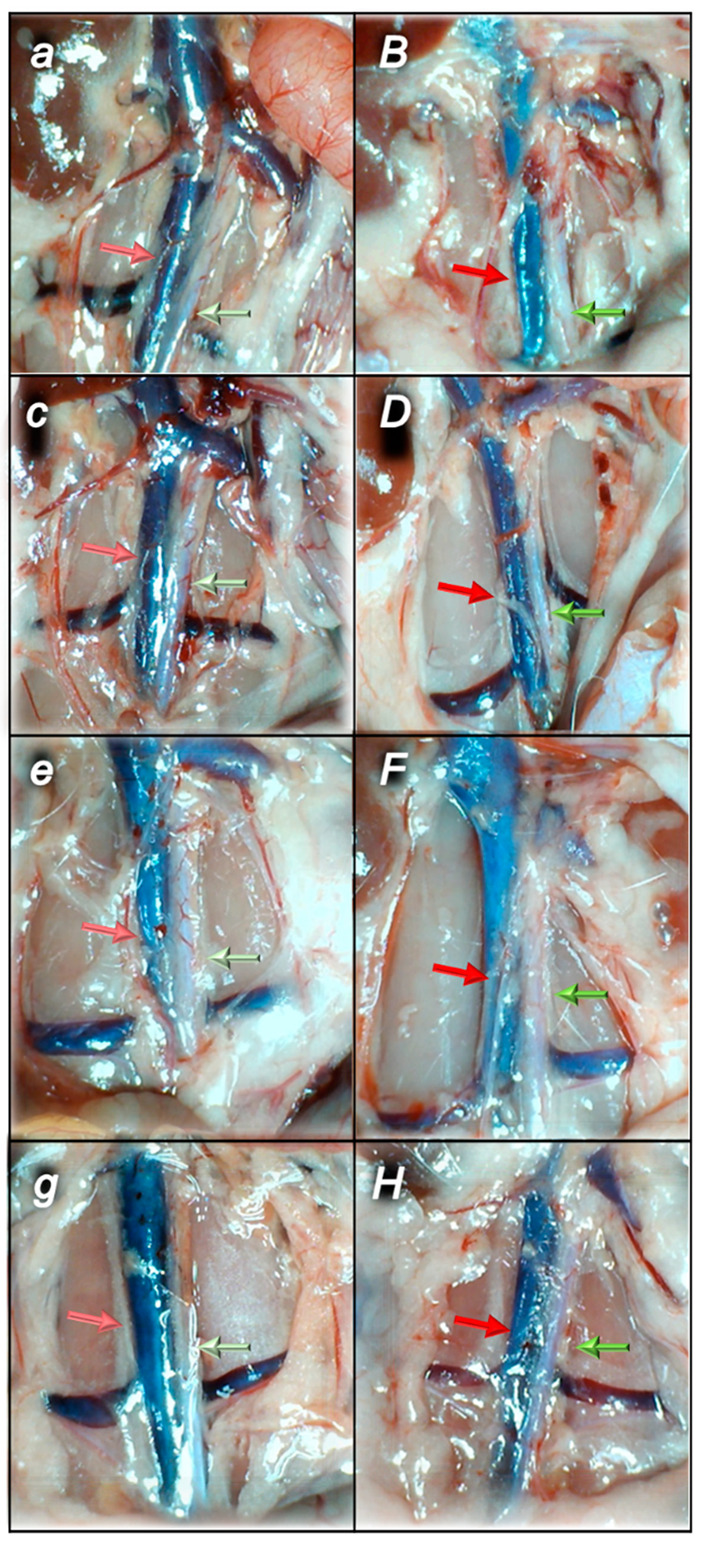
Illustrative presentation of the inferior caval vein (red arrows) and abdominal aorta (green arrows) in the sotalol control rats (small letters, light arrows) and in BPC 157-treated rats (capitals, dark arrows) assessed at 15 min (***a***,***B***), 90 min (***c***,***D***) and 180 min (***e***,***F***,***g***,***H***) following application of sotalol. Medication was given as an early application at 5 min after sotalol (***a***,***B***,***c***,***D***,***e***,***F***), or as delayed post-treatment (***g***,***H***) (at 90 min and assessment at 180 min sotalol-time), saline or BPC 157 therapy given intragastrically.

**Figure 4:**
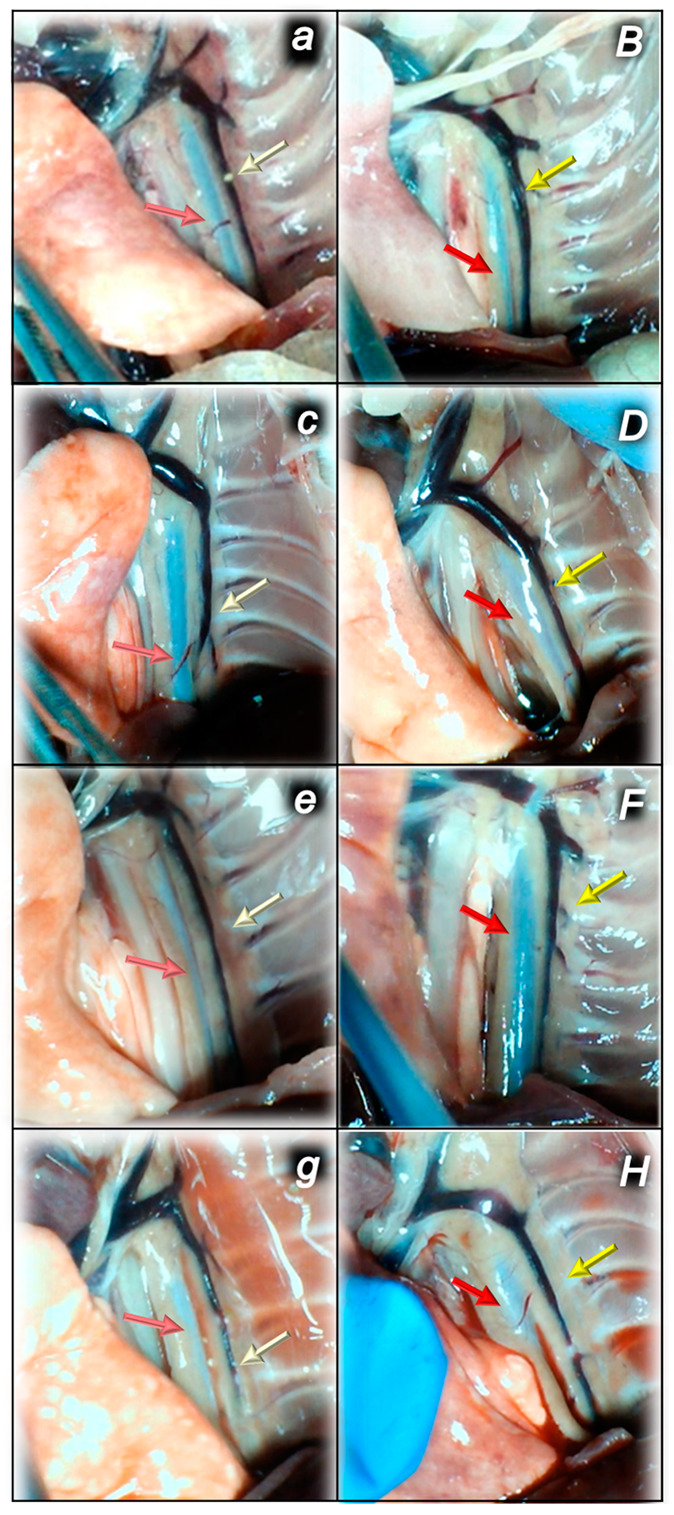
Illustrative presentation of the azygos vein (yellow arrows) and aorta (red arrows) in the sotalol control rats (small letters, light arrows) and in BPC 157-treated rats (capitals, dark arrows) assessed at 15 min (***a***,***B***), 90 min (***c***,***D***) and 180 min (***e***,***F***,***g***,***H***) following application of sotalol. Medication was given as an early application at 5 min after sotalol (***a***,***B***,***c***,***D***,***e***,***F***), or as delayed post-treatment (***g***,***H***) (at 90 min and assessment at 180 min sotalol-time), saline or BPC 157 therapy given intragastrically.

**Figure 5:**
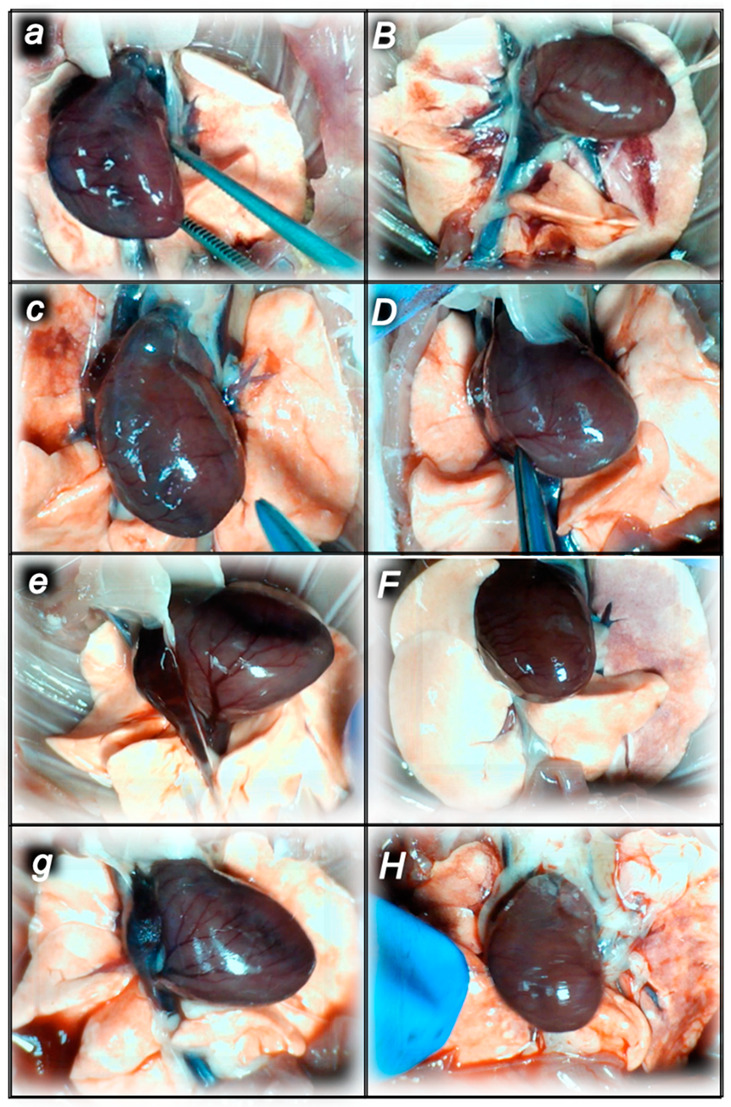
Illustrative presentation of the heart in the sotalol control rats (small letters) and in BPC 157-treated rats (capitals) assessed at 15 min (***a***,***B***), 90 min (***c***,***D***), and 180 min (***e***,***F***,***g***,***H***) following application of sotalol. Medication was given as an early application at 5 min after sotalol (***a***,***B***,***c***,***D***,***e***,***F***), or as delayed post-treatment (***g***,***H***) (at 90 min and assessment at 180 min sotalol-time), saline or BPC 157 therapy given intragastrically.

**Figure 6:**
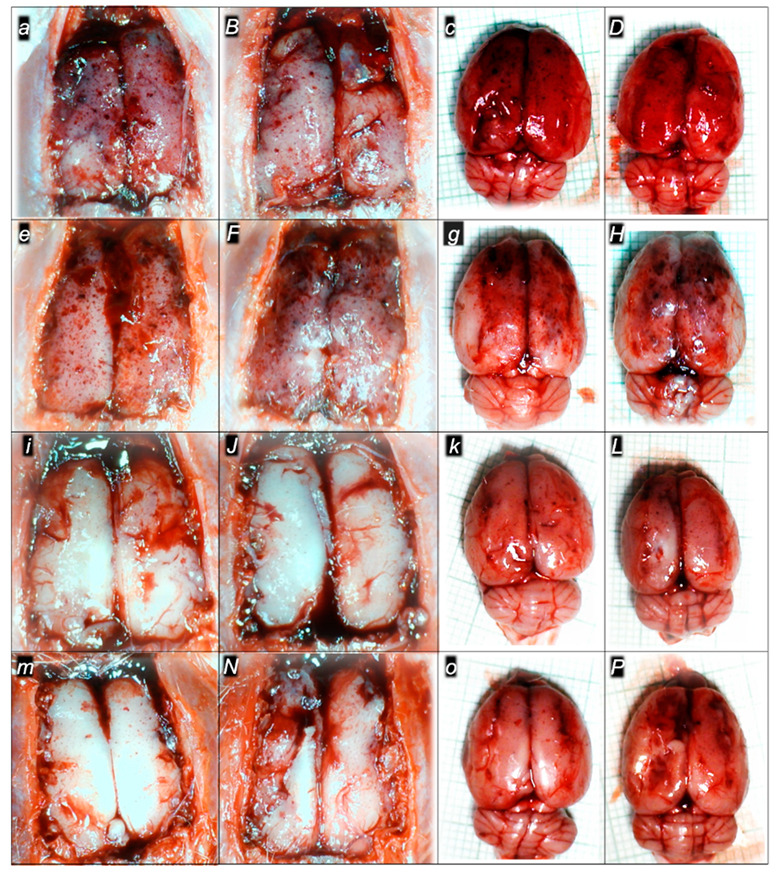
Illustrative gross presentation of the brain in the sotalol control rats (small letters) and in BPC 157-treated rats (capitals). (i) with medication given at 5 min sotalol-time assessed at 15 min sotalol-time before sacrifice (***a***,***B***) and after sacrifice (***c***,***D***), 90 min sotalol-time before sacrifice (***e***,***F***) and after sacrifice (***g***,***H***), and 180 min sotalol-time before sacrifice (***i***,***J****)* and after sacrifice (***k***,***L***). (ii) with medication given as delayed post-treatment at 90 min and assessment at 180 min sotalol-time, before sacrifice (***m***,***N***) and after sacrifice (***o***,***P***). Saline or BPC 157 therapy was given intragastrically.

There were increases in the relative volume of the congested vessels (superior mesenteric vein and inferior caval vein, from the trapped volume, congested liver and lung), dilated heart and collapsed vessels (azygos vein not functioning), and swollen brain (Table 1, Figure 2, Figure 3, Figure 4, Figure 5 and Figure 6). These might grossly appear as inactivation of the collateral pathway, inability to compensate for major vessel failure, and blood stasis observable with progressing thrombosis. Contrarily, along with attenuation/elimination of the blood pressure disturbances and thrombosis (note, congestion of the major veins, i.e., inferior caval vein and superior mesenteric vein, largely counteracted), peripherally and centrally, BPC 157 increased the azygos vein’s relative volume. Thus, prompt direct blood delivery from the inferior caval vein to the left superior caval vein might occur to re-establish blood flow (heart with normal presentation). Finally, as additional proof, the brain swelling and increased volume (associated with considerable brain injuries) were rapidly counteracted by BPC 157 administration and induced a considerable decrease toward normal brain presentation.

#### 2.1.4. Heart and ECG Disturbances

**Figure 7:**
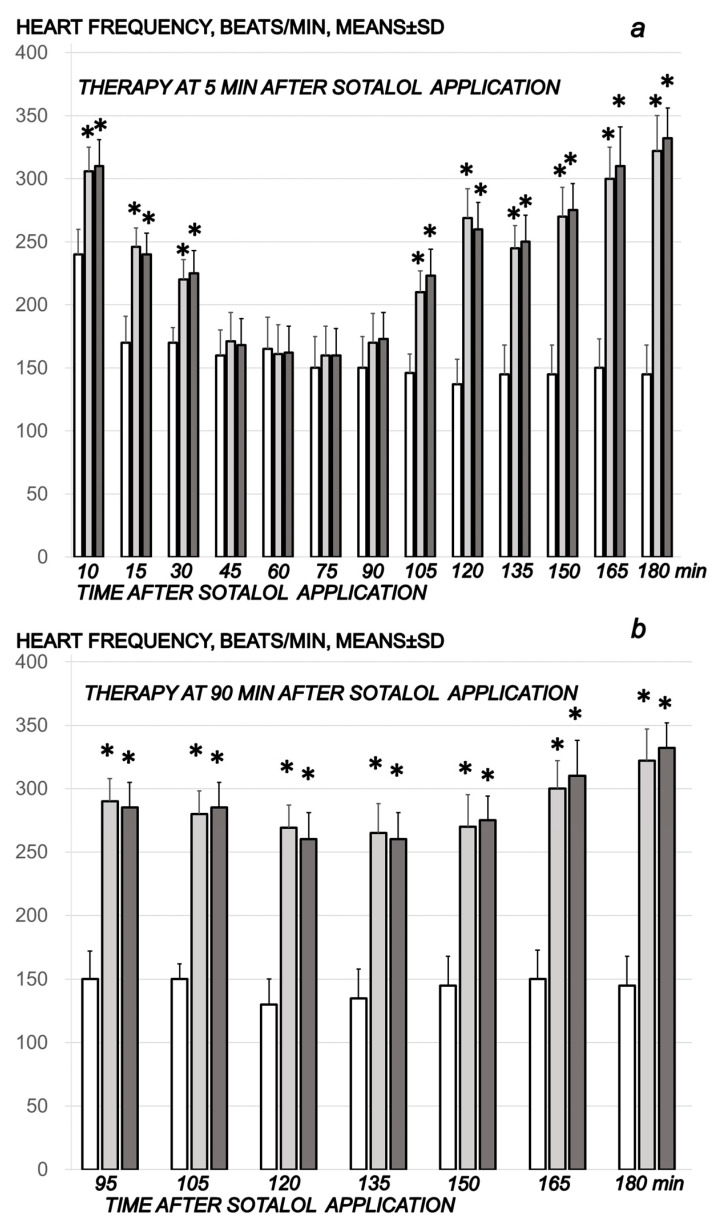
ECG assessment (***a***,***b***). Heart frequency, beats/min. Therapy included BPC 157 (10 μg/kg (light gray bars) or 10 ng/kg (dark gray bars)) or saline (5 mL/kg) (white bars) intragastric application. They were given at 5 min following sotalol and assessed at 5 min thereafter, and then at 15 min intervals until the end of the experiment (180 min sotalol-time) (***a***). As a delayed regimen (***b***), the therapy was given as an intragastric administration at 90 min sotalol-time and assessed at 5 min thereafter, and then at 15 min intervals until the end of the experiment (180 min sotalol-time). Means ± SD, * *p* ˂ 0.05, at least, vs. control.

**Figure 8:**
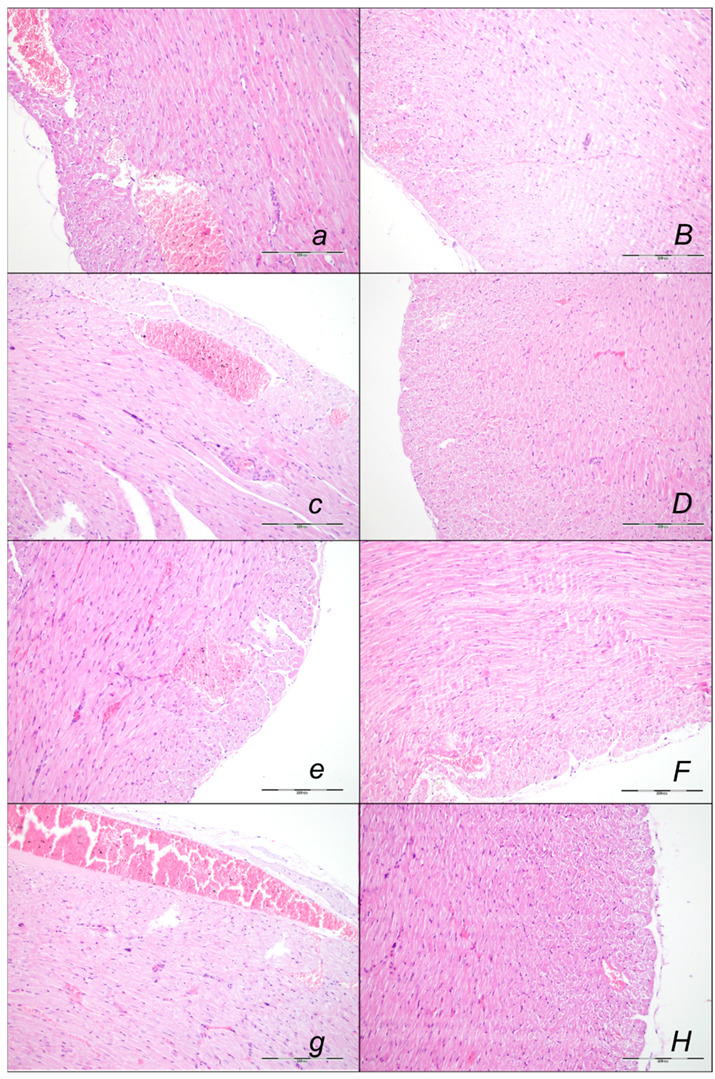
Illustrative presentation of the heart histological features (HE staining; magnification 200×; scale bar 200 μm) in the sotalol, control rats (small letters) and BPC 157-treated rats (capitals), assessed at 15 min (***a***,***B***), 90 min (***c***,***D***) and 180 min (***e***,***F***,***g***,***H***) following application of sotalol. Medication was given as an early application at 5 min after sotalol (***a***,***B***,***c***,***D***,***e***,***F***), or as delayed post-treatment (at 90 min and assessment at 180 min sotalol-time) (***g***,***H***), saline (***a***,***c***,***e***,***g***) or BPC 157 therapy (***B***,***D***,***F***,***H***) given intragastrically. Sotalol-rats treated with saline consistently exhibited marked dilatation of coronary veins and arteries, as well as myocardial vessels (***a***,***c***,***e***,***g***). Sotalol-rats treated with BPC 157 consistently exhibited either no dilatation of the coronary veins (***B*,*D*,*H***) (periods relative to therapy application 0–15 min, 0–90 min, 90–180 min) or discrete dilatation (***F***) (0–180 min).

Commonly, the sotalol procedure induced continuous bradycardia (Figure 7), while the prolonged PQ prolonged and QTc intervals were absent. With BPC 157 therapy, the counteraction of the sotalol-induced bradycardias might be a particular point. BPC 157 therapy might counteract bradycardias, either applied in the early or late course. However, the attenuated bradycardias persisted. This counteracting effect, providing both short-lasting bradycardias (BPC 157 given at 5 min sotalol-time) and long-lasting bradycardias (BPC 157 given at 90 min sotalol-time), occurred along with counteraction of the myocardial congestion (Figure 8, Table 2) and dilation (Figure 5, Table 1) and thrombosis development (Figure 1). 

### 2.2. A Perilous Syndrome Occurred Peripherally

#### 2.2.1. Heart Lung, Liver, Kidney, and Gastrointestinal Lesions

**Figure 9:**
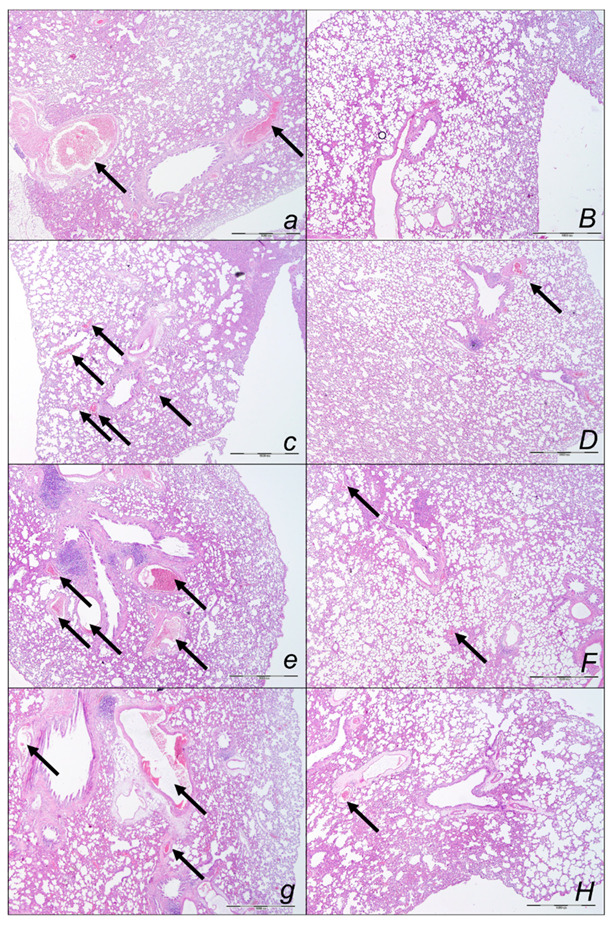
Illustrative lung congestion microscopic presentation. (HE staining; magnification 40×; scale bar 1000 μm) in sotalol, control rats (small letters) and BPC 157-treated rats (capitals), assessed at 15 min (***a***,***B***), 90 min (***c***,***D***), and 180 min (***e***,***F***,***g***,***H***) following application of sotalol. Medication was given as an early application at 5 min after sotalol (***a***,***B***,***c***,***D***,***e***,***F***), or as delayed post-treatment (at 90 min and assessment at 180 min sotalol-time) (***g***,***H***), saline (***a***,***c***,***e***,***g***) or BPC 157 therapy (***B***,***D***,***F***,***H***) given intragastrically. Sotalol-rats treated with saline consistently exhibited a marked dilatation of larger blood vessels (black arrows) and septal capillaries net (***a***,***c***,***e***,***g***). Sotalol-rats treated with BPC 157 consistently exhibited either no dilatation of the larger blood vessels and septal capillaries (***B***) (period relative to therapy application 0–15 min sotalol-time) or discrete dilatation of larger blood vessels (***D***,***F***,***H***) (periods relative to therapy application 0–90 min, 0–180 min, 90–180 min sotalol-time).

**Figure 10:**
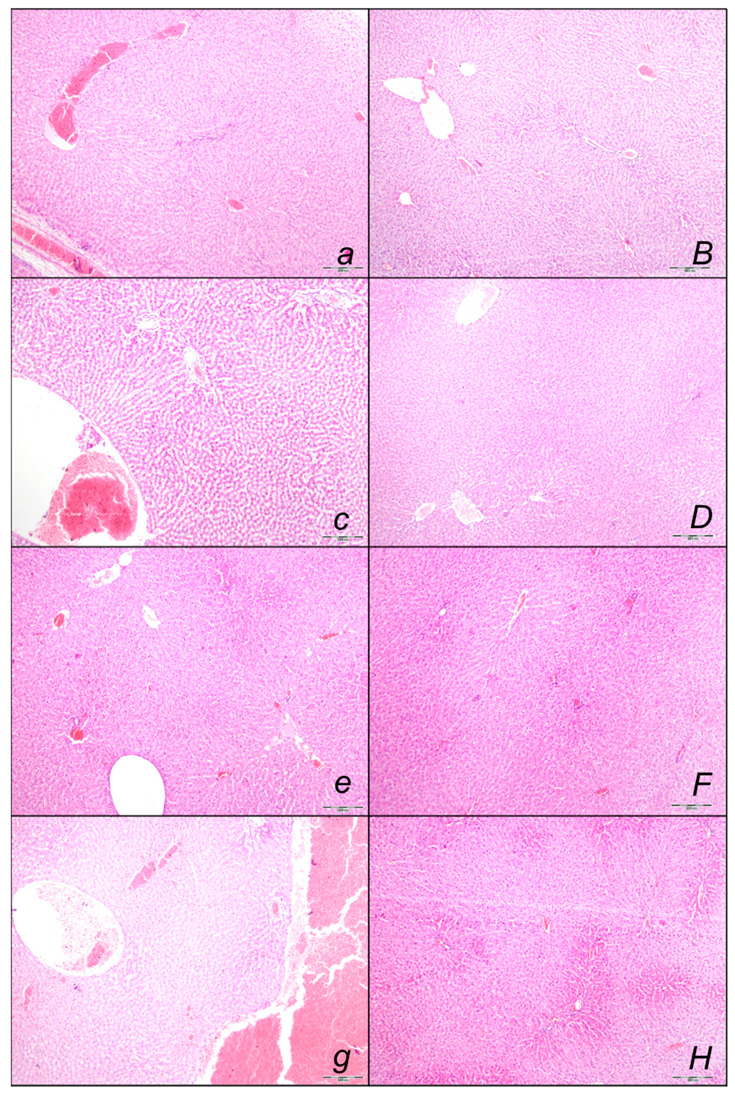
Illustrative liver tissue congestion microscopic presentation (HE staining; magnification 100×; scale bar 200 μm) in the sotalol, control rats (small letters) and BPC 157-treated rats (capitals), assessed at 15 min (***a***,***B***), 90 min (***c***,***D***) and 180 min (***e***,***F***,***g***,***H***) following application of sotalol. Medication was given as an early application at 5 min after sotalol (***a***,***B***,***c***,***D***,***e***,***F***), or as delayed post-treatment (at 90 min and assessment at 180 min sotalol-time) (***g***,***H***), saline (***a***,***c***,***e***,***g***) or BPC 157 therapy (***B***,***D***,***F***,***H***) given intragastrically. Sotalol-rats treated with saline consistently had a marked dilatation of the blood vessels in the portal tract, sinusoids, and central veins (***a***,***c***,***e***,***g***). Sotalol-rats treated with BPC 157 consistently exhibited either no dilatation (***B***,***D***,***H***) (period relative to therapy application 0–15 min, 0–90 min, 90–180 min sotalol-time) or discrete dilatation of blood vessels within central veins (***F***) (period relative to therapy application, 0–180 min sotalol-time).

**Figure 11:**
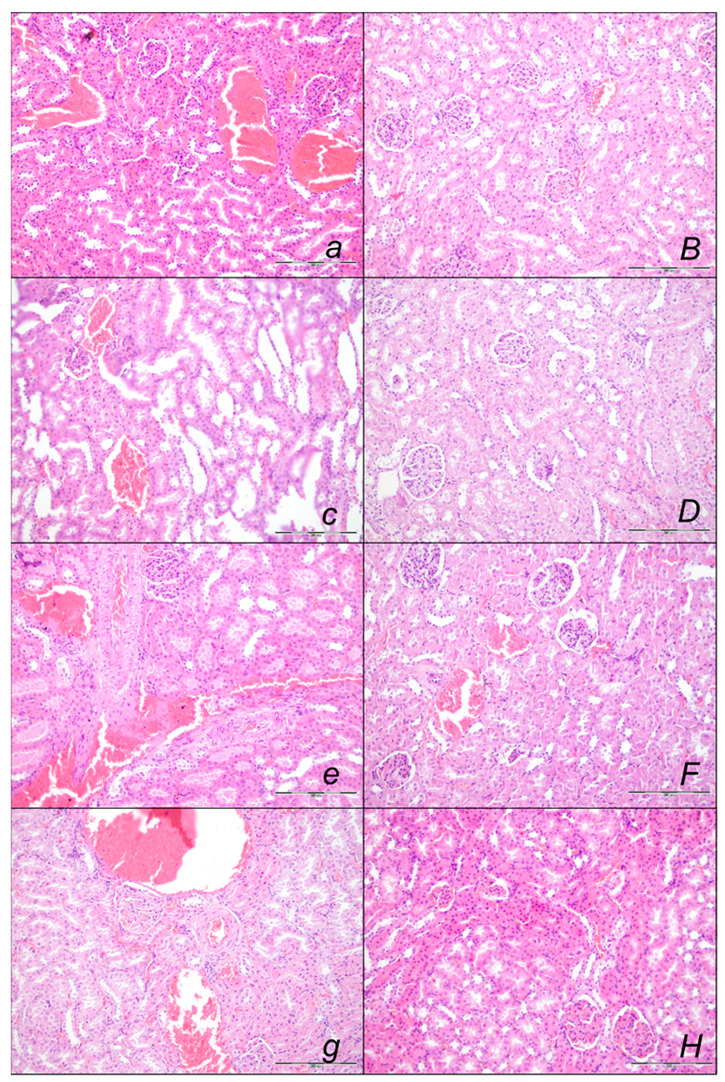
Illustrative kidney tissue congestion microscopic presentation (HE staining; magnification 200×; scale bar 200 μm) in the sotalol, control rats (small letters) and BPC 157-treated rats (capitals), assessed at 15 min (***a***,***B***), 90 min (***c***,***D***) and 180 min (***e***,***F***,***g***,***H***) following application of sotalol. Medication was given as an early application at 5 min after sotalol (***a***,***B***,***c***,***D***,***e***,***F***), or as delayed post-treatment (at 90 min and assessment at 180 min sotalol-time) (***g***,***H***), saline (***a***,***c***,***e***,***g***) or BPC 157 therapy (***B***,***D***,***F***,***H***) given intragastrically. Sotalol-rats treated with saline consistently had a marked dilatation of the terminal cortical blood vessels and glomerular capillary loop (***a***,***c***,***e***,***g***). Sotalol-rats treated with BPC 157 consistently exhibited either no dilatation (***B***,***D***,***H***) (period relative to therapy application 0–15 min, 0–90 min, 90–180 min sotalol-time) or discrete dilatation of cortical vessels (***F***) (period relative to therapy application, 0–180 min sotalol-time).

**Figure 12:**
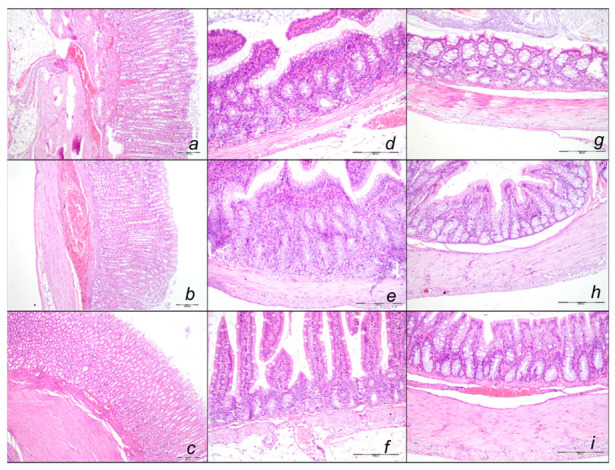
Illustrative stomach (***a****–**c***), small intestine (***d****–**f***), and colon (***g***,***h***,***i***) congestion microscopic presentation (HE staining; magnification 100× (***a–c***), scale bar 200 μm; magnification 200× (***d****–**i***), scale bar 200 μm) in the sotalol control rats assessed following application of sotalol, given therapy application at 15 min (***a***,***d***,***g***) (period 0–15 min), and at 180 min (***b***,***e***,***h***) (period 0–180 min) or at 180 min (***c***,***f***,***i***) (period 90–180 min). Saline medication was given as an early application immediately after sotalol (***a***,***b***,***d***,***e***,***g***,***h***), or as delayed post-treatment (at 90 min and assessment at 180 min sotalol-time) (***c***,***f***,***i***). Marked dilatation of submucosal blood vessels and moderate dilatation of intramucosal blood vessels occurred in the stomach, and small and large intestine.

**Figure 13:**
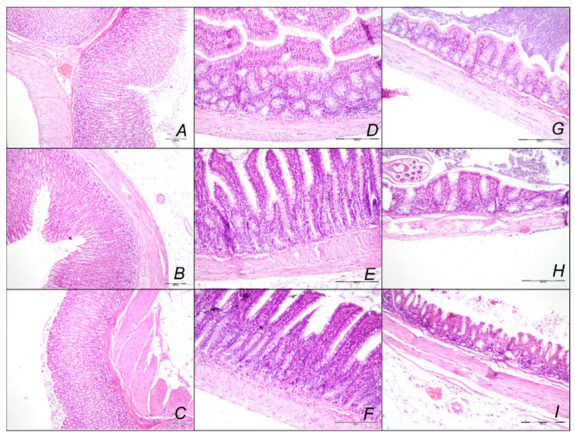
Illustrative presentation in the sotalol BPC 157-treated rats of the stomach (***A****–**C***), small intestine (***D****–**F***), and colon (***G****–**I***) congestion microscopic presentation (HE staining; magnification 100× (***A****–**C***), scale bar 200 μm; magnification 200× (***D****–**I***), scale bar 200 μm) assessed following application of sotalol, given therapy application at 15 min (***A***,***D***,***G***) (period 0–15 min), and at 180 min (***B***,***E***,***H***) (period 0–180 min), or at 180 min (***C***,***F***,***I***) (period 90–180 min). BPC 157 medication was given as an early application immediately after sotalol (***A***,***B***,***D***,***E***,***G***,***H***), or as delayed post-treatment (at 90 min and assessment at 180 min sotalol-time) (***C***,***F***,***I***). Only minor dilatation of submucosal blood vessels occurred in the stomach, with no dilatation of intestinal blood vessels.

Indicatively for a common clue that might be failed (i.e., intracranial (superior sagittal sinus), portal, and caval hypertension, and aortal hypotension, progressed thrombosis, peripherally and centrally, failed collateral recruitment), all of the sotalol regimens converge to the similar organ lesion (Table 2, Figure 8, Figure 9, Figure 10, Figure 11, Figure 12 and Figure 13). Thereby, the reduced severity of lesions by BPC 157 therapy may be seen as part of the cause-consequence therapeutic course, the recovered vascular (activation of the collateral pathway, azygos vein direct flow delivery) and heart function, along with the reduced intracranial (superior sagittal sinus), portal, and caval hypertension, and reduced aortal hypotension and immediate impact of the activated collateral pathway. In addition, in the rats challenged with sotalol, similar beneficial effects were noted with BPC 157 therapy applied intragastrically during the early or late sotalol course. 

##### Heart Lesions

Marked myocardial congestion affecting coronary veins and arteries, as well as myocardial vessels, was consistently found in the controls at the end of all of the assessed periods (Figure 8, Table 2). 

In contrast, BPC 157 might largely counteract sotalol-course. In BPC 157-treated rats there were no changes after 15 min and 90 min sotalol-time, and only mild congestion of coronary veins at 180 min sotalol-time. In addition, a marked reversal of the lesion, even in the advanced stage, might be consistently evidenced. A delayed BPC 157 regimen, given at 90 min sotalol-time, showed no myocardial changes thereafter at 180 min sotalol-time. 

##### Lung Lesions

Marked lung parenchyma congestion of larger blood vessels and septal capillaries consistently occurred in sotalol-control rats throughout the whole experiment (Figure 9, Table 2). This sotalol-damaging course was largely counteracted with BPC 157 therapy. No congestion of the lung occurred at 15 min sotalol-time, while only discrete dilatation of larger blood vessels occurred at latter periods. Especially, a marked reversal of the lesion, even in the advanced stage, might occur with a delayed BPC 157 regimen, given at 90 min sotalol-time and assessed at 180 min sotalol-time. 

##### Liver Lesions

Marked liver lesions, and marked congestion of the blood vessels in the portal tracts, sinusoids, and central veins occurred in controls at all intervals (Figure 10, Table 2). In contrast, BPC 157 might largely counteract sotalol-course. In BPC 157-treated rats, there were no changes after 15 and 90 min sotalol-time, and only discrete dilatation of blood vessels within central veins at 180 min sotalol-time. In addition, a marked reversal of the lesion, even in the advanced stage, goes with a delayed BPC 157 regimen, given at 90 min sotalol-time, and no liver changes thereafter at 180 min sotalol-time. 

##### Kidney Lesions

Marked kidney lesions, marked dilatation, and congestion of blood vessels as well as glomerular capillary loop occurred in all controls at all intervals following sotalol (Figure 11, Table 2). In contrast, BPC 157 might largely counteract sotalol-course. In BPC 157-treated rats given at 5 min sotalol-time, there were with no changes after 15 min, and 90 min sotalol-time, and only mild dilatation and congestion of blood vessels at 180 min sotalol-time. In addition, a marked reversal of the lesion, even in the advanced stage, goes with a delayed BPC 157 regimen, given at 90 min sotalol-time, and no kidney changes thereafter at 180 min sotalol-time. 

##### Stomach, Small Intestine, and Colon Lesions

Without therapy, hemorrhagic stomach lesions occurred with marked congestion of submucosal blood vessels and moderate dilatation of intramucosal blood in the stomach, small intestinal and colonic wall in controls with sotalol regimen at 15 min, 90 min, 180 min, sotalol-time (Figure 12, Table 2). In contrast, BPC 157 might largely counteract sotalol-course (Figure 13, Table 2). In BPC 157-treated rats, the sotalol-course occurred limited to only minor congestion of submucosal blood vessels in the stomach with no congestion of mucosal intestinal blood vessels. Thus, there was a marked reversal of the lesion development with the early application and an evident counteraction of the advanced stage with the delayed therapy application. 

### 2.3. A Perilous Syndrome Occurred Centrally

#### Brain Lesions of Cerebral and Cerebellar Cortex, Hypothalamus, and Hippocampus

**Figure 14:**
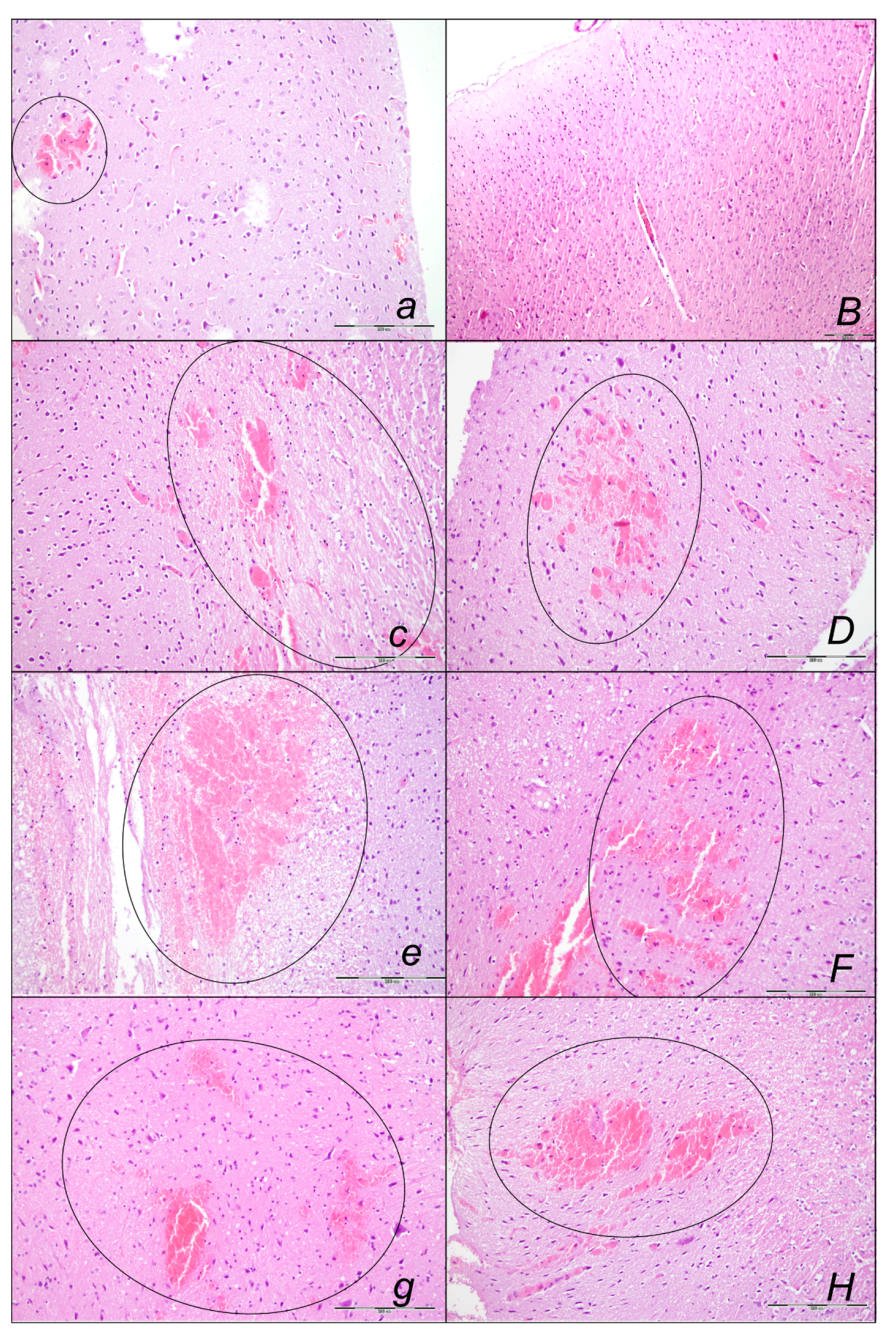
Illustrative the brain hemorrhage microscopic presentation (HE staining; magnification 100×; scale bar 200 μm) in the sotalol, control rats (italic small letters) and BPC 157-treated rats (italic capitals), assessed at 15 min (***a***,***B***), 90 min (***c***,***D***) and 180 min (***e***,***F***,***g***,***H***) following application of sotalol. Medication was given as an early application immediately after sotalol (***a***,***B***,***c***,***D***,***e***,***F***), or as delayed post-treatment (application at 90 min and assessment at 180 min sotalol-time) (***g*,*H***), saline (***a***,***c***,***e***,***g***) or BPC 157 therapy (***B***,***D***,***F***,***H***) given intragastrically. Sotalol-rats treated with saline consistently had pronounced edema, congestion, and areas of intracerebral hemorrhage (***a***,***c***,***e***,***g***) with findings of multifocal hemorrhagic areas (***g***) (marked area) in the brain tissue affecting the deeper cortical area and white matter of the brain (***e***). Sotalol-rats treated with BPC 157 had mild edema and congestion of brain tissue. No hemorrhage was noted at 15 min sotalol-time (***B***) (period relative to therapy application 0–15 min). A lesser area of intracerebral hemorrhagic appeared at 90 min and 180 min sotalol-time affecting superficial, cortical brain area (***D***,***F***,***H***) (periods relative to therapy application 0–90 min, 0–180 min, 90–180 min).

**Figure 15:**
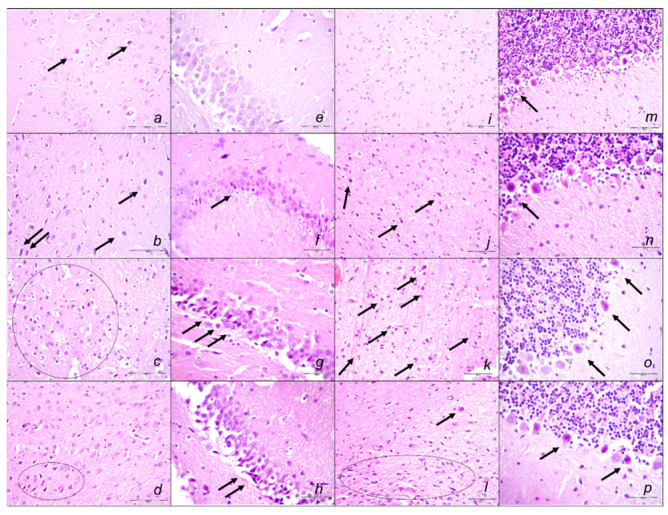
Illustrative brain karyopyknotic cell microscopic presentation (HE staining; magnification 400× (***a****–**d***), scale bar 100 μm; magnification 600× (***e****–**p***), scale bar 50 μm) in the sotalol control rats assessed following application of sotalol, given therapy application at 15 min (***a***,***e***,***i***,***m***) (period 0–15 min), at 90 min (***b***,***f***,***j***,***n***) (period 0–90 min) and at 180 min (***c***,***g***,***k***,***o***) (period 0–180 min) or at 180 min (***d***,***h***,***l***,***p***) (period 90–180 min). Saline medication was given as an early application immediately after sotalol (***a***,***b***,***d***,***e***,***g***,***h***), or as delayed post-treatment (at 90 min and assessment at 180 min sotalol-time) (***d***,***h***,***l***,***p***). An increased number of karyopyknotic cells was found in all four areas of the brain (cerebrum, hippocampus, hypothalamus, cerebellum) (black arrows and marked areas). An increased number of karyopyknotic cells was shown in the cerebrum and cerebellum with an early regimen at the end period of 15, 90, and 180 min (***a****–**d***) sotalol-time. In the hippocampus and hypothalamus, no karyopyknotic cells were observed with an early regimen at the end period of 30 min sotalol-time. An increased number of karyopyknotic cells was found in these two areas of the brain at the end period of 90 and 180 min sotalol-time.

**Figure 16:**
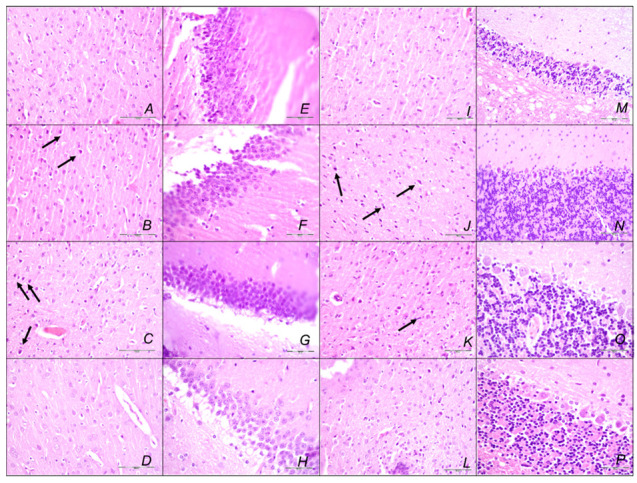
Illustrative brain karyopyknotic cell microscopic presentation (HE staining; magnification 400× (***A****–**D***), scale bar 100 μm; magnification 600× (***E****–**P***), scale bar 50 μm) in the sotalol BPC 157-treated rats assessed following application of sotalol, given therapy application at 15 min (***A***,***E***,***I***,***M***) (period 0–15 min), at 90 min (***B***,***F***,***J***,***N***) (period 0–90 min) and at 180 min (***C*,***G***,***K***,***O*****) (period 0–180 min) or at 180 min (***D***,***H***,***L***,***P***) (period 90–180 min). BPC 157 medication was given as an early application at 5 min after sotalol (***A***,***B***,***D***,***E***,***G***,***H***), or as delayed post-treatment at 90 min sotalol-time and assessment at 180 min sotalol-time (***D***,***H***,***L***,***P***). No karyopyknotic cells were observed in all four areas of the brain (cerebrum, hippocampus, hypothalamus, and cerebellum) with early regimen at the end period of 15 min (***A***,***E***,***I***,***M***) (period 0–15 min), and delayed regimen at 180 min sotalol-time (***D***,***H***,***L***,***P***) (period 90–180 min). Hippocampus (**E**–**H**) and cerebellum showed no karyopyknotic cell (***M****–**P***) with early regimen at the end period of 90 min (period 0–90) and 180 min (period 0–180 min) sotalol-time. Rare karyopyknotic cells were found in the cerebrum (***B***,***C***) and hypothalamus (***J***,***K***) with an early regimen at the end period of 90 min (period 0–90) and 180 min (period 0–180 min) sotalol-time.

Without therapy, the sotalol noxious course was highly deteriorating, with pronounced edema and congestion in the brain tissue at 15, 90, and 180 min sotalol-time, initially affecting the cerebrum and cerebellum, later also the hippocampus and hypothalamus (Table 3, Figure 14, Figure 15 and Figure 16). Illustratively, intracerebral hemorrhage initially limited to the neocortical area at 15 min, and at 90 min sotalol-time was timely progressing also to the corpus callosum, amygdala, thalamus, and striatum at 180 min sotalol-time (Figure 14, Table 3). Meantime, the depth of the hemorrhage increased three or six times, and the diameter of the hemorrhage increased over five times. Contrarily, BPC 157 rats exhibited only mild edema and congestion in the brain tissue in all intervals and all regimens (µg, ng, given at 5 min or given at 90 min sotalol-time), and hemorrhage, absent for a long time, reached at 180 min sotalol-time only a third of control hemorrhage depth and half of control hemorrhage diameter. Evidently, multifocal hemorrhage did not occur. Thus, there might be a counteraction of the lesions’ development, as well as the reversal of the already advanced lesions. 

Marked karyopyknosis occurred in all four areas of the brain in control animals (Figure 15, Table 3). They exhibited karyopyknosis and degeneration of Purkinje cells of the cerebellar cortex, and a progression from mild to marked karyopyknosis of cortical neurons and pyramidal cells of the hypothalamus. These were all attenuated in BPC 157-treated rats (Figure 16, Table 3). Besides, controls exhibited an increased number of karyopyknotic cells in two areas of the brain (cerebrum and cerebellum) in all intervals. Noteworthy, the hippocampus and hypothalamus were spared for the initial 15 min sotalol-time, while an increased number of karyopyknotic cells occurred there at 90 min and 180 min sotalol-time. BPC 157-treated rats exhibited all four areas of the brain (cerebrum, hippocampus, hypothalamus, and cerebellum) with no karyopyknotic cells at the 15 min sotalol-time. Interestingly, a similar presentation appeared with the late application at 90 min sotalol-time assessed at 180 min sotalol–time (period 90–180 min). Only rare karyopyknotic cells were found in the cerebrum and hypothalamus with an early regimen at 90 min sotalol-time (period 0–90 min) and 180 min sotalol-time (period 0–180 min). 

Thus, these results consistently revealed the beneficial effects of the BPC 157 therapy in the sotalol-induced heart failure and counteraction of the concomitant pathology, peripherally and centrally. Counteraction of sotalol-occlusion/occlusion-like syndrome was as a whole. 

## 3. Discussion

Sotalol-occlusion/occlusion-like syndrome can be the final endpoint for resolving mentioned discordances in the sotalol and antiarrhythmics effects [51,52,53,54,55,56,57,58,59,60,61,62]). Consequently, counteraction by BPC 157 therapy may be important. 

The sotalol-occlusion/occlusion-like syndrome, widely presented peripherally and centrally, specifies the responsibility of the effects of the sotalol [1,2,3], and the class II and class III antiarrhythmic and non-selective beta-blocking activity [1,2,3]. Each of the described endpoints of the occlusion/occlusion-like syndrome may prove the concept. The interconnected may be the vascular and multiorgan failure, intracranial (superior sagittal sinus) hypertension, portal, and caval hypertension, and aortal hypotension; progressive venous and arterial thrombosis peripherally and centrally, failed major veins (congested (i.e., inferior caval vein, and superior mesenteric vein) and/or collapsed (i.e., azygos vein)) and ECG disturbances (bradycardias), as rapidly acting Virchow triad circumstances [8,9,10,11,12,13,14,15,16,17,18,19,20,21,22]. The correspondence with the so far reported occlusion/occlusion-like syndromes emphasizes congruent occlusion/occlusion-like syndromes as a large multicausal class effect. Consequently, we can combine the class II and class III antiarrhythmic and non-selective beta-blocking activity [1,2,3] and several major vessel occlusions, peripherally and centrally [11,12,13,14,15,16], and other similar procedures [17,18,19,20,21,22]. Thus, these wide effects envisaged an actual general point where causatively the class II and class III antiarrhythmic and non-selective beta-blocking activity [1,2,3] might also be included. This might also be a special point with more general significance since both isoprenaline [22] and sotalol (as recently did neuroleptics, domperidone, and amphetamine) [19] might share the alike occlusion/occlusion-like syndrome [8,9,10,11,12,13,14,15,16,17,18,19,20,21,22].

Illustrating the sotalol arrhythmias, each of the organ lesions, as before in described occlusion/occlusion-like syndromes [11,12,13,14,15,16,17,18,19,20,21,22], may represent a harmful endpoint in interconnected cause-consequence relations with the noted sotalol bradycardia course. The brain (intracerebral and intraventricular hemorrhage), heart (congestion and infarctions), lung (hemorrhage), and congestion in the liver, kidney, and gastrointestinal tract, may be also related to the sotalol bradycardia course and vice versa. Likewise, as before in the counteraction of described occlusion/occlusion-like syndromes [11,12,13,14,15,16,17,18,19,20,21,22], this should characterize counteraction by BPC 157 therapy that consistently occurred.

Thereby, the counteraction of the sotalol-induced bradycardias might be a particular point in relation to the counteraction of the whole sotalol-occlusion/occlusion-like syndrome. BPC 157 therapy might counteract bradycardias, either applied in the early or late course. However, the attenuated bradycardias persisted. Thus, it seems that the antiarrhythmic and entire beneficial effect of BPC 157, consistently achieved [8,9,10,11,12,13,14,15,16,17,18,19,20,21,22], is due also to its particular effect on blood vessels. As noted before [8,9,10,11,12,13,14,15,16,17,18,19,20,21,22], upgrading the minor vessel to take over and compensate for the dysfunction of the disabled major vessel was achieved. Consequently, unlike congested or collapsed vessels, the activated collateral pathways (i.e., activated azygos vein direct blood flow delivery; inferior caval vein, and superior mesenteric vein close to normal vein presentation) might be essential [8,9,10,11,12,13,14,15,16,17,18,19,20,21,22]. The immediately increased capability of the blood vessel (i.e., rat thoracic aorta) to function even in the worst circumstances was directly demonstrated by Fourier transform infrared spectroscopy studies. There was a rapid change in the lipid contents and protein secondary structure conformation produced instantly by BPC 157 therapy [71]. Thereby, peripherally, along with previous findings [8,9,10,11,12,13,14,15,16,17,18,19,20,21,22], we noted eliminated portal and caval hypertension and attenuated aortal hypotension. Likewise, centrally, there was attenuated intracranial (superior sagittal sinus) hypertension (i.e., the upgraded venous system might ascertain the recovered capability to drain venous blood adequately for a given cerebral blood inflow rapidly normalizing venous pressures and intracranial hypertension) [11,12,13,14,15,16,17,18,19,20,21,22]. In support, the sotalol-rats with BPC 157 therapy might be capable to sustain the harmful condition of the bradycardias and non-selective beta-blocking activity without major adverse effects. Illustratively, BPC 157 therapy might eliminate otherwise regular sotalol-induced heart dilatation, and myocardial congestion affecting coronary veins, arteries, and myocardial vessels. Likewise, the extent and depth of the otherwise pronounced intracerebral hemorrhage involving larger areas of brain tissue were markedly attenuated. Similarly, in the lung parenchyma, the BPC 157 rats had no congestion of larger blood vessels and septal capillaries. Further, BPC 157 might eliminate and/or markedly attenuate liver, kidney, and gastrointestinal congestion and gross hemorrhagic gastric lesions. Progressing venous and arterial thrombosis was eliminated, and thereby, stasis was reversed, and Virchow triad reversed (and thereby attenuated/eliminated both hemorrhage and thrombosis), both peripherally and centrally, as in the previous studies [8,9,10,11,12,13,14,15,16,17,18,19,20,21,22].

Thus, the recovery of occlusion/occlusion-like syndrome of the sotalol-rats and the recovered azygos vein as a rescuing pathway and direct blood flow delivery occurred in particular in sotalol-rats treated by BPC 157. These appeared to be fully comparable with the achieved recovery of the occlusion/occlusion-like syndrome in other noxious conditions [8,9,10,11,12,13,14,15,16,17,18,19,20,21,22]. Thus, it may be that BPC 157 may fully exert its beneficial effect and avoid the consequences through an innate cytoprotective activity and vascular recovery [1,2,3,4], bringing this contention of arrhythmias into the cytoprotection issue and novel common therapy solution. Therefore, we suggested such cytoprotective effects [19] (i.e., BPC 157 therapy instantly produced rapidly changing lipid contents and protein secondary structure conformations in rat vessels (Fourier-transform infrared spectroscopy), lack of cell death [71]). Consequently, there is the increased capability of the vessel to function even in the worst circumstances [71]. Along with these, receptor blockade/stimulation and increased neurotransmission might remain unchanged but harmless [19]. In analogy, such recovery occurred even during the similar worst conditions that would otherwise be fatal and could be not removed [8,9,10,11,12,13,14,15,16,17], i.e., permanent occlusion of the major vessel [8,9,10,11,12,13,14,15,16,17], or persistently maintained severe intra-abdominal hypertension, grade III and grade IV, and persistent mechanical compression of all blood vessels [21]. Thus, as in other occlusion/occlusion-like studies [8,9,10,11,12,13,14,15,16,17,18,19,20,21,22], in sotalol-rats, the biventricular failure was effectively eliminated and/or markedly attenuated, by both peripheral and central beneficial particular action, despite potential bradycardia. Supporting this, as mentioned above, hemorrhage was simultaneously counteracted (i.e., intracerebral hemorrhage) and thrombosis was abolished, peripherally and centrally, counteracting the heart failure as a whole. Thereby, it seems that the advancing Virchow triad circumstances [8,9,10,11,12,13,14,15,16,17,18,19,20,21,22] might also be causatively resolved in the sotalol-treated rats. This particular cytoprotective effect (i.e., cytoprotection not related to any receptor but to the innate endothelium function maintenance as such [23,24]) may back particular antidysrhythmic potential against multicausal pathology noted before (digitalis [63], hyperkalemia [64], hypokalemia [65], succinylcholine [66], bupivacaine [67], lidocaine [68], and neuroleptics [69]) and recently in occlusion/occlusion-like syndromes [8,9,10,11,12,13,14,15,16,17,18,19,20,21,22]. Note, occlusion/occlusion-like syndromes presented a variety of arrhythmias, i.e., tachyarrhythmias, bradyarrhythmias, dominant ST-elevation, and prolonged QTc intervals (but also shortened QTc intervals) and asystole, depending on the injurious agent or procedure, but invariably counteracted by BPC 157 therapy [8,9,10,11,12,13,14,15,16,17,18,19,20,21,22]. 

This specific vascular action might occur as a possible modulatory effect [4,5,6,7]. Illustratively, BPC 157 might particularly combine the maintained thrombocyte function without affecting coagulation pathways [35,38,39], particular wound healing ability [72], and maintained NO-system function as a whole [31,32,33,34,35,36,37]. Thrombocyte function combines aggregometry and thromboelastometry studies [39]. BPC 157 wound healing starts from blood vessel rupture [72] and includes an innate distinctive effect on all four major events in clot formation and dissolution [72]. With BPC 157, the release of NO on its own [33,34] signifies the counteraction of NOS-blocker L-NAME-induced hypertension and pro-thrombotic effect, and counteraction of NOS-substrate L-arginine-induced hypotension and anti-coagulant effect [33,35]. Indicatively, it might affect the VEGFR2-Akt-eNOS signaling pathway without the need for other known ligands or shear stress and might exert control of the vasomotor tone through the activation of the Src-Caveolin-1-eNOS pathway [36,37]. Likewise, there were modulatory effects on the prostaglandins-system; BPC 157 counteracted NSAIDs-toxicity [73], counteracted bleeding and thrombocytopenia [35,38,39], and as membrane stabilizer counteracted leaky gut syndrome [25]. This may be particular effect in cardiac muscle, as the gap junction plays a pivotal role in electrical cell-to-cell coupling and impulse propagation between cells [74]. Accordingly, there is interaction with many other molecular pathways [25,30,36,37,40,41,42,43,44,45,46,47]. Thereby, there was also a counteraction of neuroleptics-, domperidone-, and amphetamine-induced occlusion/occlusion-like syndrome as modulatory effects on the dopamine system [19]. Accordingly, there may be in a similar way a counteraction of the isoprenaline-induced occlusion/occlusion-like syndrome [22], and now, a counteraction of sotalol-induced occlusion/occlusion-like syndrome. These may be regarded as similar modulatory effects on the adrenergic (beta) system.

Finally, there is the sotalol-occlusion/occlusion-like syndrome using a particular high sotalol protocol, 80 mg/kg intragastrically, rarely used [50], still markedly below LD50 in rats [75] as proof of the concept’s applicability. In our view, this higher regimen can be more suited than the much lower dose regimens regularly focused on cardiac events. Since the very beginning [76,77] in various species (mostly in canine models [52,53,54,55,56] but also in other species (i.e., rats, mice) [78,79,80]), regimens were mostly below 10 mg/kg as in those presented later [81,82]. These more regular doses (i.e., [83]) in regimens being accommodated to the translational human regular regimens, however, may drop even much below the requested range in humans [84,85] as translational research and drug development when back-translating the human results to rats requires dividing the rat dose by 6.2 or multiplying by 0.16 [86]. Besides, large variations in the dose regimens depending on the induced arrhythmias (i.e., 0.5 mg/kg–35 mg/kg) [87] may hamper the conclusive dosage range in animal experiments. Likewise, patients’ regular 80-, 160-, and 240-mg tablets, initial dosing with 80 mg twice daily, daily dosages between 160 and 320 mg or 480 mg, may reach dosages as high as 640 mg/d [84,85]. Contrarily, the use of the consistently higher range may approach the worst circumstances, severe sotalol intoxication with suicidal attempts [88,89], and emphasize in sotalol rat research the consistently wide range of findings in the occlusion/occlusion-like syndrome [11,12,13,14,15,16,17,18,19,20,21,22]. This would illustrate the possible importance (i.e., the innate significance of sotalol bradycardias, while QT-interval prolongation may occur later [86]). Thus, we may argue resolving following the vascular and multiorgan failure for identifying and managing theoretical strengths for resolving the earliest period even in the worst circumstance (i.e., severe sotalol intoxication with suicidal attempts [88,89]). Thereby, applicable in extreme circumstances, these may be a therapy regimen likely resolving also more regular sotalol application, and circumstances and disturbances thereof. Alternatively, by dividing the rat dose [87], the original higher doses in rats are close to those used in the patients, as opposed to the lower regimens, which may be below human regimens [84,85].

Thereby, the shared vascular, endothelium lesions, and shared cardiac lesions noted with sotalol [84,85] might be analogous to these findings in the present study. There might also be a specific effect (i.e., inhibition of potassium ions efflux by sotalol-mediated blockage of the potassium channels) [90], as BPC 157 might counteract the arrhythmias and other adverse effects in a particular way. This might be seen in rats with hyperkalemia or hypokalemia and might affect potassium channels in HEK293 cells in either hyperkalemic or hypokalemic conditions [64,65]. 

## 4. Materials and Methods

### 4.1. Animals 

Male Albino Wistar rats, 12-week-old, 200 g body weight, bred in-house at the Animal Pharmacology Facility, School of Medicine, Zagreb, Croatia (registered with the Veterinary Directorate (Reg. No: HR-POK-007)), randomly assigned at 6 rats/group/interval were used in all experiments. Rats were acclimated for five days and randomly assigned to their respective treatment groups; housed in polycarbonate (PC) cages (identified with dates, number of study, group, dose, number, and sex of each animal) at 20–24 °C, relative humidity of 40–70% and noise level 60 dB, illumination 12 h per day (fluorescent lighting), standard good laboratory practice (GLP) diet and fresh water ad libitum. Procedures were along with standard operating procedures (SOPs) of the Animal Pharmacology Facility, and the European Convention for the Protection of Vertebrate Animals used for Experimental and other Scientific Purposes (ETS 123). This study was approved by the local Ethics Committee. Ethical principles of the study complied with the European Directive 010/63/E, the Law on Amendments to the Animal Protection Act (Official Gazette 37/13), the Animal Protection Act (Official Gazette 135/06), the Ordinance on the protection of animals used for scientific purposes (Official Gazette 55/13), Federation of European Laboratory Animal Science Associations (FELASA) recommendations, and the recommendations of the Ethics Committee of the School of Medicine, University of Zagreb. The experiments were assessed by observers blinded as to the treatment.

### 4.2. Drugs

Stable gastric pentadecapeptide BPC 157 (GEPPPGKPADDAGLV, molecular weight 1419; Diagen, Slovenia), a partial sequence of the human gastric juice protein BPC, which is freely soluble in water at pH 7.0 and in saline, was prepared as a peptide with 99% high-performance liquid chromatography (HPLC) purity, with 1-des-Gly peptide being the main impurity. The BPC 157 dose and application regimens (10 µg or 10 ng/kg given as an intragastric administration, were as described previously (i.e., without the use of a carrier or peptidase inhibitor) (for review see, i.e., [11,12,13,14,15,16,17,18,19,20,21,22]). Sotalol was commercially purchased (Sigma, Aldrich, St. Louis, MO, USA). 

### 4.3. Experimental Protocol

In deeply anesthetized rats (injected 40 mg/kg thiopental (Rotexmedica, Trittau, Germany) and 10 mg/kg diazepam (Apaurin; Krka, Slovenia) intraperitoneally), complete calvariectomy was performed, and to induce rapid vascular failure and concomitant general syndrome, we applied intragastrically sotalol 80 mg/kg, and rats were sacrificed at 15, 90 or 180 min thereafter. 

Rats received therapy BPC 157 (10 µg or 10 ng/kg) or saline (5 mL/kg) (controls) as an early intragastric regimen at 5 min upon sotalol, or as delayed post-treatment, at 90 min after sotalol. 

After complete calvariectomy, recordings of brain swelling (before the procedure, after sotalol, after therapy application, and before sacrifice) followed the procedure previously used in our vascular studies [7,8,9,10,11,12,13,14,15,16,17,18,19,20]. Calvariectomy procedure included medially to the superior temporal lines and temporalis muscle attachments, 6 burr holes drilled in three horizontal lines (just basal from the posterior interocular line (two rostral burr holes); just rostral to the lambdoid suture (and transverse sinuses) on both sides (two basal burr holes); inline between the basal and rostral burr holes (two middle burr holes)). Rats were laparatomized before sacrifice for the corresponding presentation of the peripheral vessels (azygos vein, superior mesenteric vein, portal vein, inferior caval vein) and corresponding organ lesions (i.e., stomach lesion). The recording was performed with a camera attached to a VMS-004 Discovery Deluxe USB microscope (Veho, Claymont, DE, USA) at the end of the experiment, and assessed as before [8,9,10,11,12,13,14,15,16,17,18,19,20,21,22]. 

### 4.4. Superior Sagittal Sinus, Portal, and Caval Vein, and Abdominal Aorta Pressure Recording

Recordings followed the procedure used and described in detail in our previous vascular studies [7,8,9,10,11,12,13,14,15,16,17,18,19,20], (deeply anesthetized rats, a cannula (BD Neoflon™ Cannula) connected to a pressure transducer (78534C MONITOR/ TERMINAL; Hewlett Packard, Palo Alto, CA, USA), inserted into the portal vein, inferior caval vein and superior sagittal sinus, as well as the abdominal aorta at the level of the bifurcation at 15, 90, or 180 min after sotalol. The superior sagittal sinus anterior part was cannulated using a Braun intravenous cannula’ then, after laparotomy, the pressure recording in the portal vein, inferior vena cava, and abdominal aorta was performed.

Accordingly [8,9,10,11,12,13,14,15,16,17,18,19,20,21,22], superior sagittal sinus pressure of −24 to −27 mmHg, portal pressure of 3–5 mmHg similar to that of the inferior vena cava, though with values at least 1 mmHg higher in the portal vein, and abdominal aorta blood pressure values 100–120 mm Hg at the level of the bifurcation, were considered as normal in healthy rats. 

### 4.5. ECG Recording

ECGs were recorded continuously in deeply anesthetized rats for all three main leads, by positioning stainless steel electrodes on all four limbs using an ECG monitor with a 2090 programmer (Medtronic, Minneapolis, MN, USA) connected to a Waverunner LT342 digital oscilloscope (LeCroy, Chestnut Ridge, NY, USA) (before the procedure, after sotalol and after therapy application, and before sacrifice). This arrangement enabled precise recordings, measurements, and analysis of ECG parameters (PQ intervals, QTc, heart frequency) as specifically described [8,9,10,11,12,13,14,15,16,17,18,19,20,21,22]. Specifically, therapy included BPC 157 (10 μg/kg or 10 ng/kg) or saline (5 mL/kg) intragastric application. They were given at 5 min following sotalol and assessed at 5 min thereafter, and then at 15 min intervals until the end of the experiment (180 min sotalol-time). As a delayed regimen, the therapy was given as an intragastric administration at 90 min sotalol-time and assessed at 5 min thereafter, and then at 15 min intervals until the end of the experiment (180 min sotalol-time). 

### 4.6. Thrombus Assessment 

Following sacrifice, the superior sagittal sinus, and peripherally the portal vein, inferior caval vein, and abdominal aorta were removed from the rats, and the clots were weighed [8,9,10,11,12,13,14,15,16,17,18,19,20,21,22].

### 4.7. Brain Volume, Heart, and Vessel Volume Presentation

We applied the procedure used in our previous vascular studies [8,9,10,11,12,13,14,15,16,17,18,19,20,21,22]. Brain volume and vessel volume and heart volume were proportional to the change in the brain or vessel or heart surface area. The presentation of the brain and peripheral vessels (superior mesenteric vein, inferior caval vein, azygos vein, and abdominal aorta) was recorded in deeply anesthetized rats, with a camera attached to a VMS-004 Discovery Deluxe USB microscope (Veho, Claymont, DE, USA). The border of the brain (or vessels, or heart) in the image was marked using ImageJ software and then the surface area of the brain (or vessels, or heart) was measured. This was done with brain (or aorta or veins) images for healthy rats, and then/or for both the control (saline) group and treated (BPC 157) group of rats at the same intervals after the application and at the time of sacrifice. The arithmetic mean of the surface areas was calculated for both groups. Then, the ratio of these two areas was calculated as ($\frac{A_{con}}{A_{bpc}}$), where Acon is the arithmetic mean brain (or veins, or aorta, or heart) area of the control group and Abpc is the arithmetic mean brain (or veins or aorta or heart) area of the treated group. Starting from the square-cube law Equations *[1]* and *[2]*, an equation for the change in brain (or veins, or aorta, or heart) volume proportional to the change in brain surface area (or veins, or aorta, or heart) *[6]* was derived. In expressions *[1]–[5]*, *l* is defined as any arbitrary one-dimensional length of the brain (for example rostro-caudal length of the brain) (or veins, or aorta, or heart), used only for defining the one-dimensional proportion (l2/l1) between two observed brains (or vessels or heart) and as an inter-factor (and because of that not measured *[6]*) for deriving final expression *[6]*. The procedure was as follows:  $A_{2}=A_{1}\times {\left(\frac{l_{2}}{l_{1}}\right)}^{2}$ *[1]* (square-cube law), $V_{2}=V_{1}\times {\left(\frac{l_{2}}{l_{1}}\right)}^{3}$ *[2]* (square-cube law), $\frac{A_{2}}{A_{1}}={\left(\frac{l_{2}}{l_{1}}\right)}^{2}$ *[3]* (from [1], after dividing both sides by A1), $\frac{l_{2}}{l_{1}}=\sqrt{\frac{A_{2}}{A_{1}}}$ *[4]* (from *[3]*, after taking the square root of both sides), $\frac{V_{2}}{V_{1}}={\left(\frac{l_{2}}{l_{1}}\right)}^{3}$ *[5]* (from *[2]*, after dividing both sides by V1), $\frac{V_{2}}{V_{1}}={\left(\sqrt{\frac{A_{2}}{A_{1}}}\right)}^{3}$ *[6]* (after incorporating expression *[4]* into Equation *[5]*).

### 4.8. Gross Assessment of Gastrointestinal Lesions 

For recording, we used a camera attached to a VMS-004 Discovery Deluxe USB microscope (Veho, Claymont, DE, USA). As described before, gross lesions in the gastrointestinal tract, in the stomach, in particular (sum of the longest diameters, mm) were assessed in deeply anesthetized rats, laparatomized before sacrifice [8,9,10,11,12,13,14,15,16,17,18,19,20,21,22].

### 4.9. Microscopy 

As described in the previous studies [7,8,9,10,11,12,13,14,15,16,17,18,19,20], evaluation was by light microscopy using an Olympus 71 digital camera and an Olympus BX51 microscope (OLYMPUS Europa SE & CO. KG). Digital images were saved as uncompressed 24-bit RGB TIFF files using the software program AnalySIS (Olympus Soft Imaging System GmbH, Munster, Germany). The field size was measured and marked with a manufacturer’s default scale bar in the software program AnalySIS (Olympus Soft Imaging System GmbH, Munster, Germany). Representative tissue specimens (i.e., the brain, liver, kidney, stomach, small and large intestine, lungs, and heart taken at the end of the experiment, fixed in 10% neutral buffered formalin (pH 7.4) at room temperature for 24 h) were embedded in paraffin, sectioned at 4 μm, stained with hemalaun and eosin (H and E). 

#### 4.9.1. Brain Histology 

As described in the previous studies [7,8,9,10,11,12,13,14,15,16,17,18,19,20], the brain was dissected according to NTP-7, at Level 3 and 6 with neuroanatomic subsites presented in certain brain sections using coronal sections with three mandatory sections. We used a semiquantitative neuropathological scoring system, and the sum of analyzed affected areas (0–4) (i) and karyopyknotic cells in the brain areas (0–4) (ii) making (i) + (ii) a combined score (0–8), as follows. (i). Specifically affected brain areas (cerebral (NTP-7, Level 3), cerebellar cortex (NTP-7, Level 6), hippocampus, thalamus, and hypothalamus (NTP-7, Level 3)) were scored (0–4), (score 0 indicates no histopathologic change), as follows. Small, patchy, complete or incomplete infarcts (≤10% of the area affected) represented score 1. Partly confluent or incomplete infarcts (20–30% of the area affected) represented score 2. Large confluent complete infarcts (40–60% of the area affected) represented score 3. In the cortex total disintegration of the tissue, in the hypothalamus, thalamus, and hippocampus large complete infarcts (˃75% of the area affected) represented score 4. (ii). Analyzed were karyopyknotic cells in the affected brain areas (0–4) (score 0 indicates no change), cerebral (NTP-7, Level 3), cerebellar cortex (NTP-7, Level 6), hippocampus, thalamus, and hypothalamus (NTP-7, Level 3) as follows: a few karyopyknotic of neuronal cells (≤20%) (score 1); patchy areas of karyopyknotic cells (50%) (score 2); more extensive karyopyknotic areas (75%) (score 3); complete infarction (100%) (score 4). Brain tissue hemorrhage was obtained by estimating a percentage of affected areas. Intraventricular hemorrhage was noted as present or absent.

We also assessed the neuronal pathological changes in acquired digital images saved as uncompressed 24-bit RGB TIFF files in the software program AnalySIS (Olympus Soft Imaging System GmbH, Munster, Germany) performing quantitative analysis of neuronal damage in the karyopyknotic areas. The neurons of the cortical cerebral, cerebellar region, hippocampus, and hypothalamus were counted in 10 different high-powered fields (HPF, 400×) and 3 to 5 serial sections of each sample were used to do the count as described [91]. The field size was 0.24 μm^2^. 

We used four criteria for the estimation of the edema: pale myelin, sieve-like appearance of myelinated areas, dilation of perivascular and pericellular spaces, and vacuolar appearance of the neuropil of gray matter. Edema was graded as heavy, moderate, slight, or no edema (score 0–3) [92]. 

#### 4.9.2. Lung Histology 

The same scoring system as in the previous studies [8,9,10,11,12,13,14,15,16,17,18,19,20,21,22] was used to grade the degree of lung injury in lung tissue analysis. Each of the features (i.e., focal thickening of the alveolar membranes, congestion, pulmonary edema, intra-alveolar hemorrhage, interstitial neutrophil infiltration, and intra-alveolar neutrophil infiltration) was scored (0–3) as absent (0) or present a mild (1), moderate (2), or severe (3) degree, and a final histology score was determined.

#### 4.9.3. Renal, Liver, and Heart Histology 

The same scoring system as in the previous studies [8,9,10,11,12,13,14,15,16,17,18,19,20,21,22] was used to grade renal (i.e., the degeneration of Bowman’s space and glomeruli, degeneration of the proximal and distal tubules, vascular congestion, and interstitial edema), liver (i.e., vacuolization of hepatocytes and pyknotic hepatocyte nuclei, activation of Kupffer cells, and enlargement of sinusoids) and heart (i.e., dilatation and congestion of blood vessels within the myocardium and coronary arteries) histology. Each specimen was scored using a scale ranging from 0–3 (0: none, 1: mild, 2: moderate, and 3: severe) for each criterion, and a final histology score was determined (0: none, 1: mild, 2: moderate, and 3: severe). 

#### 4.9.4. Gastrointestinal Histology 

As in previous studies [8,9,10,11,12,13,14,15,16,17,18,19,20,21,22], we used a histologic scoring scale adapted from Chui and coworkers [93] for the stomach tissue damage scoring 0–5 (normal to severe) in three categories (mucosal injury, inflammation, hyperemia/hemorrhage) for a total score of 0 to 15, as described by Lane and coworkers [94]. Illustratively, the assessment included morphologic features of mucosal injury (i.e., different grades of epithelial lifting, villi denudation, and necrosis), inflammation (i.e., focal to diffuse according to lamina propria infiltration or subendothelial infiltration), and hyperemia/hemorrhage (i.e., focal to diffuse according to lamina propria or subendothelial localization). 

### 4.10. Statistical Analysis

Statistical analysis was performed by parametric one-way analysis of variance (ANOVA), with the Newman-Keuls post-hoc test or the non-parametric Kruskal-Wallis test and subsequently, the Mann-Whitney U test to compare groups. Values are presented as the mean ± standard deviation (SD) and as the minimum/median/maximum. To compare the frequency difference between groups, the chi-squared test or Fischer’s exact test was used. *p* < 0.05 was considered statistically significant.

## 5. Conclusions

In conclusion, also with class II and class III antiarrhythmic and non-selective beta-blockade, BPC 157 vascular recovery therapy was able to provide adequate compensation (i.e., activation of collateral pathways to reestablish blood flow), both rapid and sustained, as demonstrated with BPC 157 therapy and counteracted sotalol-induced occlusion/occlusion-like syndrome as whole [8,9,10,11,12,13,14,15,16,17,18,19,20,21,22]. Contrarily, if not corrected for an innate inability to react, with beta-blockade-induced failure, damaged endothelium function would inevitably initiate rapidly a perilous course in occlusion/occlusion-like syndrome, further progressing (i.e., as noted in the brain, with hypothalamic and hippocampal lesions that appeared in the later course). The sotalol-occlusion/occlusion-like syndrome, innate vascular and multiorgan failure, and heart failure might be comparable to the one induced by major vessel occlusion (ligation) as well as upon other alike noxious procedures (i.e., alcohol, lithium, isoprenaline, bile duct occlusion, and maintained high intra-abdominal pressure) [8,9,10,11,12,13,14,15,16,17,18,19,20,21,22]. Given overwhelming thrombosis, and thereby stasis, peripherally and centrally, class II and class III antiarrhythmic and non-selective beta-blockade might be considered all as multiple occlusion/occlusion-like syndrome [8,9,10,11,12,13,14,15,16,17,18,19,20,21,22]. On the other hand, the BPC 157 bypassing key therapy [8,9,10,11,12,13,14,15,16,17,18,19,20,21,22] might be regarded as part of the general beneficial pleiotropic effect (as part of the cytoprotection background that might be obtained also by the per-oral application used in the present study) [4,5,6,7]. Thus, with BPC 157 therapy occurring also during beta-blockade, the counteraction of the concomitant severe vessel and multiorgan failure syndrome might be perceived as a network of mutually supporting evidence tightly interconnected. Therefore, the counteraction of heart failure as a whole along with the counteraction of the severe lesions in the brain, lung, liver, kidney, and gastrointestinal tract, almost annihilated thrombosis, and hemorrhage, peripherally and centrally consistently occurred [8,9,10,11,12,13,14,15,16,17,18,19,20,21,22]. Also, these might be all consistently seen as a network of the evidence for the physiologic significance of the revealed BPC 157/vascular-system interplay (i.e., BPC 157 was found in situ hybridization and immunostaining studies in humans to be largely distributed in tissues [95] and may have additional physiologic regulatory roles [72,95]). Based on the alike beneficial effects, similar importance was suggested also for other species (i.e., birds [96] and insects [97,98]). Moreover, there is also a very safe BPC 157 profile (i.e., no adverse effects in clinical trials (ulcerative colitis, phase II), and in toxicological studies, lethal dose (LD1) could be not achieved) (for review see [1,3,4,5,6,7,25,32,72,73,95]), a point recently confirmed in a large study conducted by Xu and collaborators [99]. 

Thus, we obtained the beneficial effects of the BPC 157 therapy in the sotalol-induced heart failure and counteraction of the concomitant pathology, peripherally and centrally, counteraction of occlusion/occlusion-like syndrome as a whole. Therefore, also confronted with the disabled beta sympathetic system, it might be that the heart failure cause-consequence circuit might occur in a multidirectional way that BPC 157 therapy might beneficially affect as a whole.

## Figures and Tables

**Table 1:** Relative volume (control/treated) (%) of the brain, heart, azygos vein, in rats at 15 min and 90 min, and 180 min following sotalol application. Therapy included BPC 157 (10 μg/kg or 10 ng/kg) or saline (5 mL/kg). They were given as an intragastric administration at 5 min following sotalol for assessment at 15 min (period 0–15 min sotalol-time), 90 min (period 0–90 min sotalol-time) and 180 min (period 0–180 min sotalol-time). As a delayed regimen (period 90–180 min sotalol-time), the therapy was given as an intragastric administration at 90 min sotalol-time for assessment at 180 min sotalol-time. Means ± SD, * *p ˂ 0.05*, at least, vs. control.

	Relative Volume (Control/Treated) (%) of the Brain, Heart, Azygos Vein, in Rats at 30 min and 90 min, and 180 min following Sotalol Application			
Periods Assessed in Relation to the Medication Application	0–15 min	0–90 min	0–180 min	90–180 min
	Relative volume (control/treated) (%) of the brain, in vivo, Means ± SD			
BPC 157 10 μg/kg	111 ± 3 *	111 ± 3 *	116 ± 3 *	110 ± 3 *
BPC 157 10 ng/kg	112 ± 3 *	112 ± 3 *	114 ± 3 *	111 ± 3 *
	Relative volume (control/treated) (%) of the heart, Means ± SD			
BPC 157 10 μg/kg	135 ± 5 *	139 ± 7 *	146 ± 6 *	139 ± 7 *
BPC 157 10 ng/kg	132 ± 6 *	142 ± 5 *	144 ± 5 *	141 ± 6 *
	Relative volume (control/treated) (%) of the inferior caval vein, Means ± SD			
BPC 157 10 μg/kg	155 ± 8 *	149 ± 9 *	156 ± 6 *	199 ± 10 *
BPC 157 10 ng/kg	151 ± 9 *	155 ± 8 *	159 ± 9 *	191 ± 11 *
	Relative volume (control/treated) (%) of the superior mesenteric vein, Means ± SD			
BPC 157 10 μg/kg	225 ± 8 *	229 ± 9 *	216 ± 9 *	289 ± 10 *
BPC 157 10 ng/kg	222 ± 9 *	227 ± 8 *	224 ± 9 *	281 ± 8 *
	Relative volume (control/treated) (%) of the azygos vein, Means ± SD			
BPC 157 10 μg/kg	35 ± 6 *	69 ± 7 *	66 ± 5 *	68 ± 7 *
BPC 157 10 ng/kg	32 ± 7 *	67 ± 8 *	62 ± 9 *	61 ± 68 *
	Relative volume (control/treated) (%) of the abdominal aorta, Means ± SD			
BPC 157 10 μg/kg	32 ± 6 *	66 ± 7 *	76 ± 8 *	43 ± 7 *
BPC 157 10 ng/kg	35 ± 7 *	63 ± 8 *	72 ± 9 *	41 ± 6 *
	Relative volume (control/treated) (%) of the brain, ex vivo, Means ± SD			
BPC 157 10 μg/kg	114 ± 3 *	110 ± 2 *	116 ± 3 *	111 ± 3 *
BPC 157 10 ng/kg	113 ± 3 *	111 ± 3 *	115 ± 3 *	112 ± 3 *

**Table 2:** Microscopic presentation of the lesions in the heart, lung, liver, kidney, stomach, small intestine, and large intestine, and gross lesions presentation in the stomach in rats at 15 min and 90 min, and 180 min following sotalol application. Therapy included BPC 157 (10 μg/kg or 10 ng/kg) or saline (5 mL/kg). They were given as an intragastric administration at 5 min following sotalol for assessment at 15 min (period 0–15 min sotalol-time), 90 min (period 0–90 min sotalol-time) and 180 min (period 0–180 min sotalol-time). As a delayed regimen (period 90–180 min sotalol-time), the therapy was given as an intragastric administration at 90 min sotalol-time for assessment at 180 min sotalol-time. Min/Med/Max, Means ± SD, * *p* ˂ 0.05, at least, vs. control.

	Microscopic Presentation of the Lesions in the Heart, Lung, Liver, Kidney, Stomach, Small Intestine, and Large Intestine, and Gross Lesions Presentation in the Stomach in Rats at 15 min and 90 min, and 180 min following Sotalol Application			
Periods Assessed in Relation to the Medication Application	0–15 min	0–90 min	0–180 min	90–180 min
	Heart (scored 0–3, Min/Med/Max)			
Saline 5 mL/kg (control)	3/3/3	3/3/3	3/3/3	3/3/3
BPC 157 10 μg/kg	0/0/0 *	0/0/0 *	0/1/1 *	0/0/0 *
BPC 157 10 ng/kg	0/0/0 *	0/0/0 *	0/1/1 *	0/0/0 *
	Lung (scored 0–3, Min/Med/Max)			
Saline 5 mL/kg (control)	3/3/3	3/3/3	3/3/3	3/3/3
BPC 157 10 μg/kg	0/0/0 *	1/1/1 *	1/1/1 *	1/1/1 *
BPC 157 10 ng/kg	0/0/0 *	1/1/1 *	1/1/1 *	1/1/1 *
	Liver (scored 0–3, Min/Med/Max)			
Saline 5 mL/kg (control)	3/3/3	3/3/3	3/3/3	3/3/3
BPC 157 10 μg/kg	0/0/0 *	0/0/0 *	0/1/1 *	0/0/0 *
BPC 157 10 ng/kg	0/0/0 *	0/0/0 *	0/1/1 *	0/0/0 *
	Kidney (scored 0–3, Min/Med/Max)			
Saline 5 mL/kg (control)	3/3/3	3/3/3	3/3/3	3/3/3
BPC 157 10 μg/kg	0/0/0 *	0/0/0 *	0/1/1 *	0/0/0 *
BPC 157 10 ng/kg	0/0/0 *	0/0/0 *	0/1/1 *	0/0/0 *
	Stomach (sum of longest diameters, mm, means ± SD)			
Saline 5 mL/kg (control)	5 ± 1	6 ± 1	5 ± 1	5 ± 1
BPC 157 10 μg/kg	0 ± 0 *	0 ± 0 *	0 ± 0 *	0 ± 0 *
BPC 157 10 ng/kg	0 ± 0 *	0 ± 0 *	0 ± 0 *	0 ± 0 *
	Stomach (scored 0–15, Min/Med/Max)			
Saline 5 mL/kg (control)	5/5/5	5/5/5	5/5/5	5/5/5
BPC 157 10 μg/kg	1/1/1 *	1/1/1 *	1/1/1 *	1/1/1 *
BPC 157 10 ng/kg	1/1/1 *	1/1/1 *	1/1/1 *	1/1/1 *
	Small intestine (scored 0–15, Min/Med/Max)			
Saline 5 mL/kg (control)	5/5/5	5/5/5	5/5/5	5/5/5
BPC 157 10 μg/kg	0/0/0 *	0/0/0 *	0/0/0 *	0/0/0 *
BPC 157 10 ng/kg	0/0/0 *	0/0/0 *	0/0/0 *	0/0/0 *
	Large intestine (scored 0–15, Min/Med/Max)			
Saline 5 mL/kg (control)	5/5/5	5/5/5	5/5/5	5/5/5
BPC 157 10 μg/kg	0/0/0 *	0/0/0 *	0/0/0 *	0/0/0 *
BPC 157 10 ng/kg	0/0/0 *	0/0/0 *	0/0/0 *	0/0/0 *

**Table 3:** Microscopic presentation of the lesions in the brain lesions in the cerebrum, cerebellum, hippocampus, and hypothalamus in rats at 15 and 90 min, and 180 min following sotalol application. Therapy included BPC 157 (10 μg/kg or 10 ng/kg) or saline (5 mL/kg). They were given as an intragastric administration at 5 min following sotalol for assessment at 15 min (period 0–15 min sotalol-time), 90 min (period 0–90 min sotalol-time) and 180 min (period 0–180 min sotalol-time). As a delayed regimen (period 90–180 min sotalol-time), the therapy was given as an intragastric administration at 90 min sotalol-time for assessment at 180 min sotalol-time. Min/Med/Max, Means ± SD, * *p ˂ 0.05*, at least, vs. control. # combined score (0–8)–semiquantitative neuropathological scoring system; the sum of affected areas with infarction and karyopyknotic cells.

	Microscopic Presentation of the Brain Lesions in the Cerebrum, Cerebellum, Hippocampus, and Hypothalamus at 15 min and 30 min, and 180 min following Sotalol Application			
Periods Assessed in Relation to the Medication Application	0–15 min	0–90 min	0–180 min	90–180 min
	**Cerebrum (scored 0–8, Min/Med/Max) #**			
Saline 5 mL/kg (control)	1/1/1	1/2/2	2/2/2	2/2/2
BPC 157 10 μg/kg	0/0/0 *	0/1/1 *	0/1/1 *	0/0/0 *
BPC 157 10 ng/kg	0/0/0 *	0/1/1 *	0/1/1 *	0/0/0 *
	Neuronal damage in the karyopyknotic areas, %, Means ± SD (10 HPF, 400×)			
Saline 5 mL/kg (control)	10 ± 10	21 ± 10	32 ± 10	29 ± 10
BPC 157 10 μg/kg	0 ± 0 *	5 ± 5 *	5 ± 5 *	0 ± 0 *
BPC 157 10 ng/kg	0 ± 0 *	5 ± 5 *	5 ± 5 *	0 ± 0 *
	Hemorrhage (% of total area), Means ± SD			
Saline 5 mL/kg (control)	5 ± 2	10 ± 4	15 ± 4	15 ± 3
BPC 157 10 μg/kg	0 ± 0 *	5 ± 1 *	10 ± 2 *	10 ± 2 *
BPC 157 10 ng/kg	0 ± 0 *	5 ± 1 *	10 ± 2 *	9 ± 2 *
	Edema (scored 0–3, Min/Med/Max)			
Saline 5 mL/kg (control)	3/3/3	3/3/3	3/3/3	3/3/3
BPC 157 10 μg/kg	1/1/1 *	1/1/1 *	1/1/1 *	1/1/1 *
BPC 157 10 ng/kg	1/1/1 *	1/1/1 *	1/1/1 *	1/1/1 *
	Cerebellum (scored 0–8, Min/Med/Max) *			
Saline 5 mL/kg (control)	0/1/1	0/1/1	0/1/1	0/1/1
BPC 157 10 μg/kg	0/0/0 *	0/0/0 *	0/0/0 *	0/0/0 *
BPC 157 10 ng/kg	0/0/0 *	0/0/0 *	0/0/0 *	0/0/0 *
	Neuronal damage in the karyopyknotic areas, %, Means ± SD (10 HPF, 400×)			
Saline 5 mL/kg (control)	9 ± 5	11 ± 5	34 ± 10	32 ± 10
BPC 157 10 μg/kg	0 ± 0 *	0 ± 0 *	0 ± 0 *	0 ± 0 *
BPC 157 10 ng/kg	0 ± 0 *	0 ± 0 *	0 ± 0 *	0 ± 0 *
	Hemorrhage (% of total area), Means ± SD			
Saline 5 mL/kg (control)	0 ± 0	0 ± 0	0 ± 0	0 ± 0
BPC 157 10 μg/kg	0 ± 0	0 ± 0	0 ± 0	0 ± 0
BPC 157 10 ng/kg	0 ± 0	0 ± 0	0 ± 0	0 ± 0
	Edema (scored 0–3, Min/Med/Max)			
Saline 5 mL/kg (control)	3/3/3	3/3/3	3/3/3	3/3/3
BPC 157 10 μg/kg	1/1/1 *	1/1/1 *	1/1/1 *	1/1/1 *
BPC 157 10 ng/kg	1/1/1 *	1/1/1 *	1/1/1 *	1/1/1 *
	Hippocampus (scored 0–8, Min/Med/Max) *			
Saline 5 mL/kg (control)	0/0/0	0/0/0	1/1/1	1/1/1
BPC 157 10 μg/kg	0/0/0	0/0/0	0/0/0	0/0/0
BPC 157 10 ng/kg	0/0/0	0/0/0	0/0/0	0/0/0
	Neuronal damage in the karyopyknotic areas, %, Means ± SD (10 HPF, 400×)			
Saline 5 mL/kg (control)	0 ± 0	10 ± 5	36 ± 5	33 ± 5
BPC 157 10 μg/kg	0 ± 0	0 ± 0 *	0 ± 0 *	0 ± 0 *
BPC 157 10 ng/kg	0 ± 0	0 ± 0 *	0 ± 0 *	0 ± 0 *
	Hemorrhage (% of total area), Means ± SD			
Saline 5 mL/kg (control)	0 ± 0	0 ± 0	0 ± 0	0 ± 0
BPC 157 10 μg/kg	0 ± 0	0 ± 0	0 ± 0	0 ± 0
BPC 157 10 ng/kg	0 ± 0	0 ± 0	0 ± 0	0 ± 0
	Edema (scored 0–3, Min/Med/Max)			
Saline 5 mL/kg (control)	2/2/2	3/3/3	3/3/3	3/3/3
BPC 157 10 μg/kg	1/1/1 *	1/1/1 *	1/1/1 *	1/1/1 *
BPC 157 10 ng/kg	1/1/1 *	1/1/1 *	1/1/1 *	1/1/1 *
	Hypothalamus (scored 0–8, Min/Med/Max) *			
Saline 5 mL/kg (control)	0/0/0	0/2/2	2/2/2	2/3/3
BPC 157 10 μg/kg	0/0/0	0/1/1 *	0/1/1 *	0/0/0 *
BPC 157 10 ng/kg	0/0/0	0/1/1 *	0/1/1 *	0/0/0 *
	Neuronal damage in the karyopyknotic areas, %, Means ± SD (10 HPF, 400×)			
Saline 5 mL/kg (control)	0 ± 0	13 ± 5	42 ± 5	39 ± 5
BPC 157 10 μg/kg	0 ± 0	5 ± 2 *	10 ± 5 *	0 ± 0 *
BPC 157 10 ng/kg	0 ± 0	6 ± 2 *	11 ± 5 *	0 ± 0 *
	Hemorrhage (% of total area), Means ± SD			
Saline 5 mL/kg (control)	0 ± 0	0 ± 0	0 ± 0	0 ± 0
BPC 157 10 μg/kg	0 ± 0	0 ± 0	0 ± 0	0 ± 0
BPC 157 10 ng/kg	0 ± 0	0 ± 0	0 ± 0	0 ± 0
	Edema (scored 0–3, Min/Med/Max)			
Saline 5 mL/kg (control)	2/2/2	3/3/3	3/3/3	3/3/3
BPC 157 10 μg/kg	1/1/1 *	1/1/1 *	1/1/1 *	1/1/1 *
BPC 157 10 ng/kg	1/1/1 *	1/1/1 *	1/1/1 *	1/1/1 *

## Data Availability

The data presented in this study are available on request from the corresponding author.
